# Real-Time Electronic Patient-Reported Outcome Monitoring With Digital Triage and Care-Team Integration in Early Breast Cancer: ENABLE Randomized Controlled Trial

**DOI:** 10.2196/95155

**Published:** 2026-10-28

**Authors:** Thomas M Deutsch, Léa L Volmer, Manuel Feisst, Marcus Heise, Kathrin Hassdenteufel, Lara M Tretschock, André Pfob, Christiane Breit, Ekaterina Martynenko, Stefan Stefanovic, Nima Ghalekhani, Stephanie Wallwiener, Eva Kantelhardt, Marlene Martin, Armin Bauer, Tjeerd M H Dijkstra, Laura L Michel, Tobias Engler, Andreas D Hartkopf, Nico Pfeifer, Sara Y Brucker, Andreas Schneeweiss, Markus Wallwiener

**Affiliations:** 1 Department of Obstetrics and Gynecology, University of Heidelberg, Heidelberg, Baden Wuerttemberg, Germany; 2 Department of Gynecology and Obstetrics, Mannheim University Hospital, University of Heidelberg, Theodor-Kutzer-Ufer 1-3, Mannheim, Baden Wuerttemberg, 68167, Germany, +49 621 383 2288; 3 Department of Womenʼs Health, University of Tübingen, Tübingen, Germany; 4 Institute of Medical Biometry, University of Heidelberg, Heidelberg, Germany; 5 Department of Gynecology, University Hospital Halle (Saale), Martin Luther University Halle-Wittenberg, Halle (Saale), Germany; 6 Institute of General Practice and Family Medicine, Medical Faculty, Martin Luther University Halle-Wittenberg, Halle (Saale), Germany; 7 Breast Center Heidelberg, Hospital St. Elisabeth, Heidelberg, Germany; 8 Department of Obstetrics and Prenatal Medicine, University Hospital Halle (Saale), Martin Luther University Halle-Wittenberg, Halle (Saale), Germany; 9 Institute of Women’s Health GmbH, Tübingen, Germany; 10 National Center for Tumor Diseases (NCT), Heidelberg, Germany; 11 Methods in Medical Informatics, Department of Computer Science, University of Tübingen, Tübingen, Germany; 12 Center for Innovative Care, University Hospital Tübingen, Tübingen, Germany; 13 Institute for Bioinformatics and Medical Informatics, University of Tübingen, Tübingen, Germany; 14 German Cancer Research Center (DKFZ), Heidelberg, Germany

**Keywords:** ePRO intervention, electronic patient-reported outcome, randomized trial, primary breast cancer, health-related quality of life, symptom monitoring

## Abstract

**Background:**

Electronic patient-reported outcomes (ePROs) can support patient-centered oncology care, but in early breast cancer, it is unclear whether real-time ePRO-triggered triage adds benefit beyond lower-intensity app-based support and scheduled ePRO collection.

**Objective:**

We tested whether adding weekly ePRO screening, rule-based triage, and care-team escalation to app-based support improves health-related quality of life (HRQoL) during systemic therapy for early breast cancer.

**Methods:**

ENABLE was a multicenter, open-label, parallel-group randomized controlled trial at 2 German university hospitals. Adults with early (stage I-III) breast cancer initiating systemic therapy were randomized 1:1 to reactive patient-reported outcome assessment (RPA) or control. Both arms received the ENABLE app with educational and organizational support and scheduled HRQoL assessments at 6 fixed time points; only RPA received weekly EuroQol Visual Analogue Scale screening (trigger: score <50 or ≥10-point decline) followed by symptom assessment, digital triage, and care-team alerts. Patient-Reported Outcomes Measurement Information System-29 was analyzed as an 8-measure HRQoL end-point family (7 domains plus pain intensity); no single domain, composite score, or binary global success rule was prespecified. Group-by-visit mixed-effects models were fitted with Benjamini-Hochberg false-discovery-rate adjustment. The recruitment period was March 22, 2021, to September 8, 2023.

**Results:**

Of 605 randomized patients, 487 entered the modified intention-to-treat analysis (RPA: n=242; control: n=245). End-of-treatment between-group differences (RPA minus control, *T*-score points, 95% CI, and adjusted *P*) were: fatigue −3.90 (95% CI −5.77 to −2.03; *P*=.004), social participation +2.84 (95% CI 1.18 to 4.49; *P*=.01), anxiety −2.10 (95% CI −3.65 to −0.55; *P*=.049), depression −1.95 (95% CI −3.25 to −0.65; *P*=.10), sleep disturbance −1.82 (95% CI −3.43 to −0.20; *P*=.051), pain interference −1.44 (95% CI −3.38 to 0.49; *P*=.18), physical function +1.33 (95% CI −0.22 to 2.88; *P*=.18), and pain intensity −0.26 (95% CI −0.73 to 0.21; *P*=.32; 0‐10 scale). All *T*-score differences were below the 5-point minimally important difference (MID). Exploratory time-to-deterioration analyses favored RPA for fatigue (hazard ratio [HR] 0.69, 95% CI 0.54‐0.89), social participation (HR 0.76, 95% CI 0.59-0.97), and depression (HR 0.66, 95% CI 0.44-0.98). Of 209 monitored RPA patients, 160 (76.6%; 95% CI 70.4‐81.8) triggered at least 1 alert. No intervention-related harms were identified.

**Conclusions:**

ENABLE tests real-time, rule-based ePRO triage with care-team escalation against an active digital comparator rather than usual care, isolating the response pathway itself; it extends this design from metastatic disease into early breast cancer and escalates to the care team rather than to the patient alone. Benefits were domain-specific and sub-MID, in anxiety, fatigue, and social participation, tempering the expectation of a global HRQoL effect. For practice, symptom-triggered triage is a supportive-care workflow tool worth embedding where alerts reach a team that acts and documents, not a stand-alone remedy for treatment-related morbidity.

## Introduction

### Background

Breast cancer is the most common cancer in women and a leading cause of cancer death worldwide [1]. Most patients with early-stage disease receive multimodal systemic therapy, and treatment has shifted from uniform protocols toward regimens tailored to tumor biology and individual risk [2]. This has improved outcomes but lengthened and intensified the treatment pathway: months of chemotherapy, surgery, radiotherapy, and often years of endocrine therapy. The associated symptom burden is substantial and is not confined to the treatment phase itself, with impairments in fatigue, sleep, and emotional and social functioning still reported years after diagnosis [3,4]. Symptom burden also has consequences for treatment delivery: delayed initiation of neoadjuvant and adjuvant chemotherapy is associated with worse survival [5,6], and patients’ own accounts show that the path toward such delays is shaped by how early symptoms and concerns are recognized and acted upon [7]. Maintaining tolerability is therefore not only a quality-of-life goal but part of delivering the planned treatment as intended.

Patient-centered care therefore requires attention not only to tumor-directed outcomes but also to patients’ preferences, values, symptoms, functioning, and lived treatment experience [8]. Patient-reported outcome measures (PROMs) provide a standardized way to capture symptom burden and health-related quality of life (HRQoL) domains that may otherwise remain underrecognized in routine oncology visits [9], covering physical, emotional, and social experiences across the cancer trajectory and helping clinical teams identify unmet needs, assess treatment tolerability, and tailor supportive interventions [3,10-15]. Collected electronically (electronic patient-reported outcomes [ePROs]), they can enable more timely monitoring, support shared decision-making, and provide structured symptom information to patients and clinicians [4,16]. The mechanisms proposed for these effects—earlier detection of deterioration, earlier supportive intervention, and better-informed consultations—depend less on the act of collecting data than on what follows once a symptom has been reported [15]. PROMs and ePROs are recommended in international clinical practice guidelines [17] and have been associated with improved symptom control and quality of care; survival effects have been reported in some trials and populations but have not been consistent across larger multicenter evaluations [13,16,18].

Despite this evidence base, important implementation questions remain. Many ePRO systems focus on scheduled symptom or HRQoL assessment, whereas fewer systems combine patient-facing monitoring with automated triage logic, tailored advice, and structured escalation to the treating care team [19,20]. Systems also differ in ways that are rarely stated explicitly: whether a predefined threshold triggers a response, who receives the signal, whether the response itself is specified rather than left to discretion, and whether it is documented. These design choices plausibly account for part of the heterogeneity in reported effects [20]. Such real-time, care team–integrated escalation is particularly relevant for early breast cancer, where the therapeutic goal differs from the metastatic setting: treatment is intensive but time-limited, dose- and schedule-dependent, and the priority is to complete it as planned [2,5,6].

Evidence on ePRO interventions in neoadjuvant and adjuvant early breast cancer remains limited. It is therefore uncertain whether benefits observed in other oncology populations translate to early breast cancer and also uncertain whether real-time triage with structured care-team escalation adds benefit beyond scheduled ePRO assessment and app-based educational support. This uncertainty justified an active digital comparator: both trial arms received the ENABLE app, educational and organizational support, and scheduled HRQoL assessments, while only the intervention arm received weekly EuroQol Visual Analogue Scale (EQ-VAS) screening, automated symptom triage, and care-team escalation. Most previous trials compared ePRO monitoring with usual care, so their effects may reflect the combined contribution of structured contact, digital information, and symptom reporting rather than the triage step alone [20,21]. An active comparator sets a higher bar and attenuates the expected effect size, but it isolates the component that would have to be built, staffed, and maintained if such a workflow was adopted in routine care.

### Objectives

The ENABLE trial was designed to test the incremental value of this reactive digital triage model during systemic therapy for early breast cancer in a real-world oncology setting. We tested the hypothesis that adding weekly EQ-VAS screening, rule-based digital triage, and care-team escalation to app-based ePRO monitoring and educational support would improve Patient-Reported Outcomes Measurement Information System-29 (PROMIS-29) HRQoL trajectories, analyzed as an 8-measure end-point family, compared with app-based ePRO monitoring and educational support alone. Treatment satisfaction assessed with the Patient Satisfaction Questionnaire Short Form (PSQ-18) was evaluated as a secondary end point. Secondary objectives were to describe intervention delivery and engagement in the intervention arm and to examine time to clinically meaningful HRQoL deterioration.

## Methods

### Trial Design

This trial is reported in accordance with the CONSORT (Consolidated Standards of Reporting Trials) 2025 statement (Checklist 1) [22] and the CONSORT-eHEALTH (Consolidated Standards of Reporting Trials of Electronic and Mobile Health Applications and Online Telehealth) extension for web-based and mobile health interventions (Checklist 2) [23]. We conducted a multicenter, randomized, unblinded, parallel-group, superiority trial (1:1) in adults initiating adjuvant, neoadjuvant, or postneoadjuvant systemic therapy for early breast cancer. Participants were centrally randomized in a 1:1 ratio to reactive patient-reported outcome assessment (RPA) or control.

### Trial Setting

The trial was planned and ethics-approved as a 3-center trial at German academic breast-cancer centers (Heidelberg, Tübingen, and Mannheim). Recruitment and follow-up took place at Heidelberg and Tübingen. Mannheim University Hospital was ethics-approved and listed in the public registry (Mannheim 2021‐544, approved March 16, 2021) but ultimately enrolled no participants and was therefore not included in the analyses. Eligible sites were academic breast-cancer centers with an established adjuvant or neoadjuvant chemotherapy service and the infrastructure to operate the ENABLE platform; the intervention was delivered by trained clinical study teams (study nurses and treating oncologists) at each site. Recruitment was clinic-based and closed to enrolled participants. Participants were consecutively screened and enrolled before treatment start. The study was not an open-access web trial with anonymous self-enrollment. Because recruitment and consent occurred through study sites, multiple identities were not a relevant concern in the way typical for open online trials.

### Trial Registration and Protocol Availability

The protocol is publicly available (*JMIR Research Protocols* 2025 [24]), and the trial is registered with the German Clinical Trials Register (DRKS00025611). The trial was registered on July 6, 2021, after enrollment of the first participant on March 22, 2021; registration was therefore retrospective. The delay was administrative and occurred during finalization of the multicenter trial documentation and public registry entry for a 3-center trial; the registry entry lists Heidelberg, Tübingen, and Mannheim, although Mannheim subsequently enrolled no participants. The delay did not involve any change to the trial design or the outcome measures. The trial design and the outcome measures had been prespecified in the submission on August 27, 2020, to the central ethics committee, which approved the trial on October 2, 2020, more than 5 months before the first participant was enrolled (S-658/2020); both dates are recorded in the registry entry itself. The protocol was amended during the trial, most substantially to increase the target sample size in October 2022; the outcome measures were not affected.

### Changes to Trial Protocol

The trial was registered retrospectively: the first participant was enrolled on March 22, 2021, and the trial was registered on July 6, 2021 (DRKS00025611); the registry entry lists Heidelberg, Tübingen, and Mannheim, although Mannheim enrolled no participants. In a change from the protocol, recruitment in the palliative setting was not pursued at the study sites because concurrent recruitment to the PRO-B trial (DRKS00024015) was ongoing; the protocol had originally allowed inclusion across settings including palliative therapy. The target sample size was increased by protocol amendment on October 27, 2022, as described under the Sample Size section; this amendment did not affect the outcome measures. The published protocol stated that the primary outcomes would be examined in an intention-to-treat analysis of all randomized individuals. The analysis reported here uses a modified intention-to-treat (mITT) population: 4 participants in whom metastatic disease was identified after randomization were excluded, and the population comprises 487 of the remaining 601 participants who provided at least 1 postrandomization assessment of the primary end point. The composition of the analysis population was determined at the analysis stage; neither the ethics-approved protocol nor the published protocol defined an analysis population beyond the sentence quoted earlier. Both the protocol and the registry name treatment satisfaction alongside HRQoL in the statement of the primary end point; treatment satisfaction (PSQ-18) is reported here as a secondary end point, consistent with the protocol’s list of secondary end points and with the instrument, timing, sample-size basis, and analysis strategy it specified (see the Outcomes section). The Benjamini-Hochberg (BH) procedure applied across the 8 PROMIS-29 measures to control the false discovery rate was chosen at the analysis stage and was not described in the protocol. The protocol designated no single PROMIS-29 domain, composite score, or binary global success rule as primary, and the primary end point is therefore reported as an 8-measure family. The protocol named 2 instruments for the assessment of patient satisfaction, the PSQ-18 and the PEF-FB-9 (Fragebogen zur Partizipativen Entscheidungsfindung). The PEF-FB-9 was not administered, and no data on it were collected. Only the PSQ-18 is reported here.

### Ethical Considerations

Central ethics approval was obtained from the ethics committee of the Medical Faculty, Heidelberg University (S-658/2020). All recruiting centers obtained local approval: ethics committee at the Medical Faculty of the Eberhard Karls University and University Hospital Tübingen (896/2020BO2) and ethics committee II of Heidelberg University, Medical Faculty Mannheim (2021‐544). The study was conducted in accordance with the Declaration of Helsinki. All participants provided written informed consent prior to enrollment, including consent for publication of anonymized data. Patient data were handled in accordance with the European General Data Protection Regulation (GDPR) and the German Federal Data Protection Act. Patient privacy and confidentiality were maintained throughout the study. No financial compensation was provided to participants. No images or other materials that could identify individual participants are included in this manuscript or its supplementary materials. The ethics committee approvals for this trial, each listing the documents submitted to the committee with their version numbers and dates, are provided as supplementary files.

### Patient and Public Involvement

No patients or members of the public were involved in the design, conduct, reporting, or dissemination plans of this study.

### Harms

Harms were not systematically ascertained through a predefined adverse-event reporting protocol, consistent with the nonpharmacological, digital nature of the intervention; no separate safety-monitoring instrument was administered. Nonetheless, no intervention-related safety concerns, privacy breaches, or clinically relevant unintended digital effects were identified during the study.

### Eligibility Criteria

Adults (≥18 years) with histologically confirmed, nonmetastatic, invasive breast cancer (stage I-III) scheduled to initiate systemic therapy in a neoadjuvant, adjuvant, or postneoadjuvant setting were consecutively screened and enrolled before treatment start at the university hospitals of Heidelberg and Tübingen (Germany). Key eligibility criteria were German language proficiency, capacity to provide written informed consent, and access to an internet-enabled device (smartphone, tablet, or computer).

### Randomization: Sequence Generation

The allocation sequence was computer-generated in R (version 3.6.3; blockrand version 1.5; R Foundation for Statistical Computing) as site-specific block-randomization lists (Heidelberg, Tübingen, and Mannheim; the Mannheim list was not used, as that site enrolled no participants) with random block sizes of 2, 4, 6, and 8, 1:1 allocation, and no stratification or minimization factors. The sequence was generated by the biostatistics unit and implemented by the study IT team in a central web portal; neither unit was involved in participant enrollment or clinical care.

### Allocation Concealment Mechanism

Allocation was concealed by the central system until assignment. Group allocation became visible to participants and site staff only after randomization within the portal.

### Implementation

Study nurses enrolled participants at each site and triggered the central assignment, without access to the random allocation sequence.

### Blinding

Due to the nature of the digital intervention, the trial was unblinded. Participants, clinical teams, outcome assessors, and data analysts were unmasked, and no masking-success assessment was performed.

### Intervention and Comparator

#### Overview

The intervention and its delivery are reported in line with the TIDieR (Template for Intervention Description and Replication) checklist [25]; the core components, provider, mode, setting, and schedule are specified below and in Figure 1.

**Figure 1.**
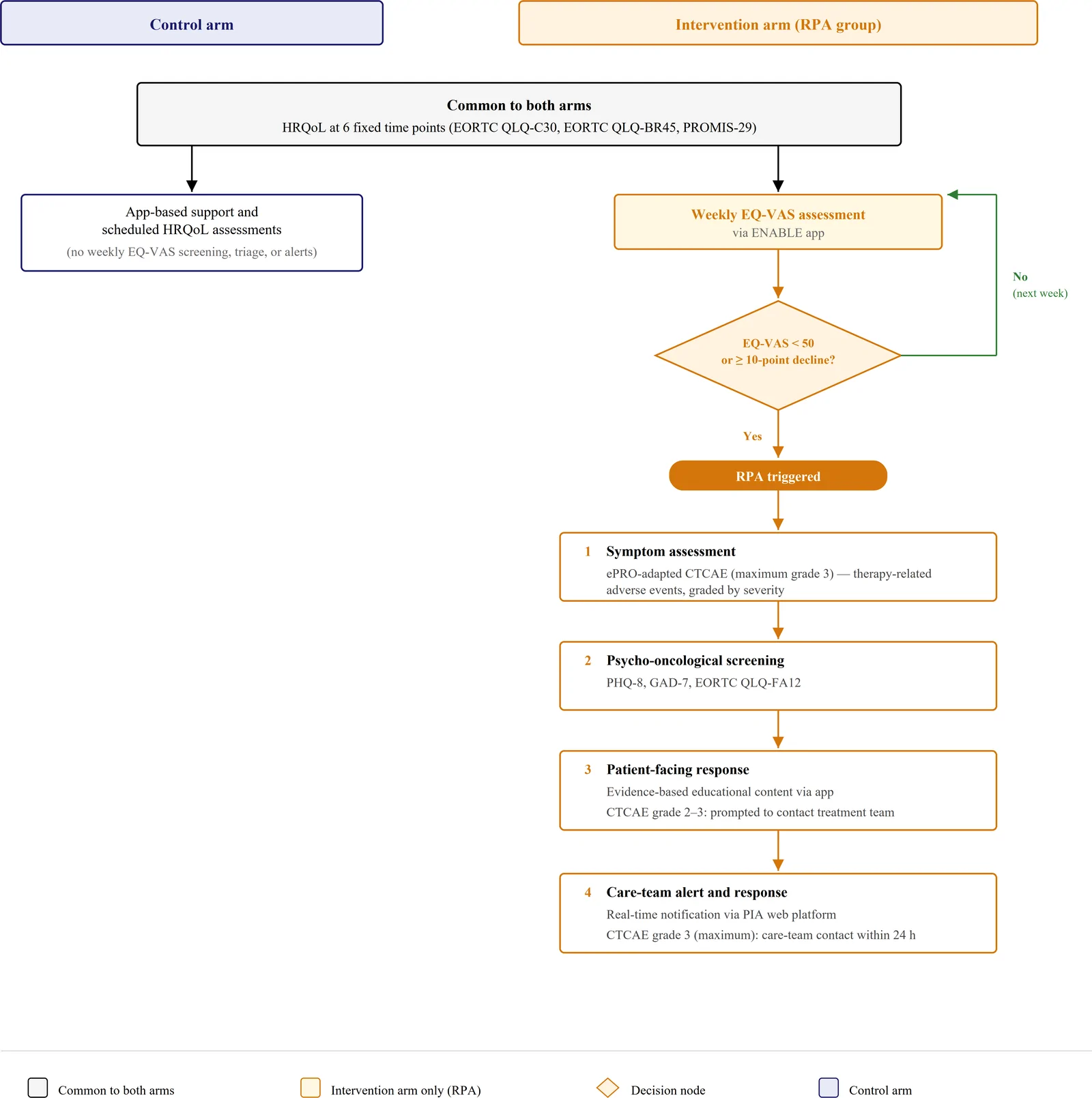
RPA—rule-based digital triage and escalation pathway with care-team integration. The RPA pathway applied in the intervention arm: a weekly EQ-VAS trigger (score <50 or ≥10-point decline) initiated symptom assessment with an ePRO-adapted CTCAE (maximum grade 3; grades 4‐5 were not used), psycho-oncological screening, patient-facing educational content (patient prompted to contact the treatment team at CTCAE grades 2‐3), and real-time care-team alerting (care-team contact within 24 hours at CTCAE grade 3). The control arm received the same app-based support and the same scheduled HRQoL assessments, without weekly EQ-VAS screening, automated triage, or care-team alerts. CTCAE: Common Terminology Criteria for Adverse Events; ePRO: electronic patient-reported outcome; EORTC QLQ-BR45: European Organisation for Research and Treatment of Cancer Quality of Life Questionnaire Breast 45; EORTC QLQ-C30: European Organisation for Research and Treatment of Cancer Quality of Life Questionnaire Core 30; EQ-VAS: EuroQol Visual Analogue Scale; EORTC QLQ-FA12: European Organisation for Research and Treatment of Cancer Quality of Life Questionnaire Fatigue 12; GAD-7: Generalized Anxiety Disorder 7-item scale; HRQoL: health-related quality of life; PROMIS-29: Patient-Reported Outcomes Measurement Information System-29; PHQ-8: Patient Health Questionnaire-8; PIA: ENABLE web-based care-team platform; RPA: reactive patient-reported outcome assessment.

#### Common to Both Arms

HRQoL was assessed at 6 fixed time points during therapy using the European Organisation for Research and Treatment of Cancer Quality of Life Questionnaire Core 30 (EORTC QLQ-C30), European Organisation for Research and Treatment of Cancer Quality of Life Questionnaire Breast 45 (EORTC QLQ-BR45), and PROMIS-29. Assessments were conducted prior to initiating systemic therapy, at 3 time points during adjuvant or neoadjuvant chemotherapy, after completion of chemotherapy, and following surgery and/or radiotherapy. For patients receiving adjuvant endocrine or postneoadjuvant therapy, assessments were distributed over 1 year (Figure 2). All participants received access to the patient-facing ENABLE native smartphone app (iOS and Android), including a personalized treatment and care plan, a content library providing self-care strategies for adverse event management, nutritional counseling, social support services, physiotherapy and exercise programs, and evidence-based educational materials displayed as a customized feed tailored to the patient’s therapy and individual needs. A calendar function displayed the patient’s current treatment status and upcoming appointments. After the final visit, patient satisfaction was assessed via the PSQ-18 (administered at the conclusion of adjuvant or neoadjuvant chemotherapy or after 1 year of endocrine or postneoadjuvant therapy). The app environment included institutional identification of participating study centers. Logos and addresses of the respective centers were displayed within the study environment accessible to enrolled participants.

Clinical teams at each site accessed a web-based care-team platform (PIA) for patient enrollment, assignment to personalized care plans, appointment scheduling, and clinical data entry. The ENABLE app and the PIA care-team platform were developed and provided by the Center for Innovative Care and the Institute of Women’s Health GmbH (Tübingen, Germany); trial data were stored and managed on a secured cloud infrastructure operated by these partners.

**Figure 2.**
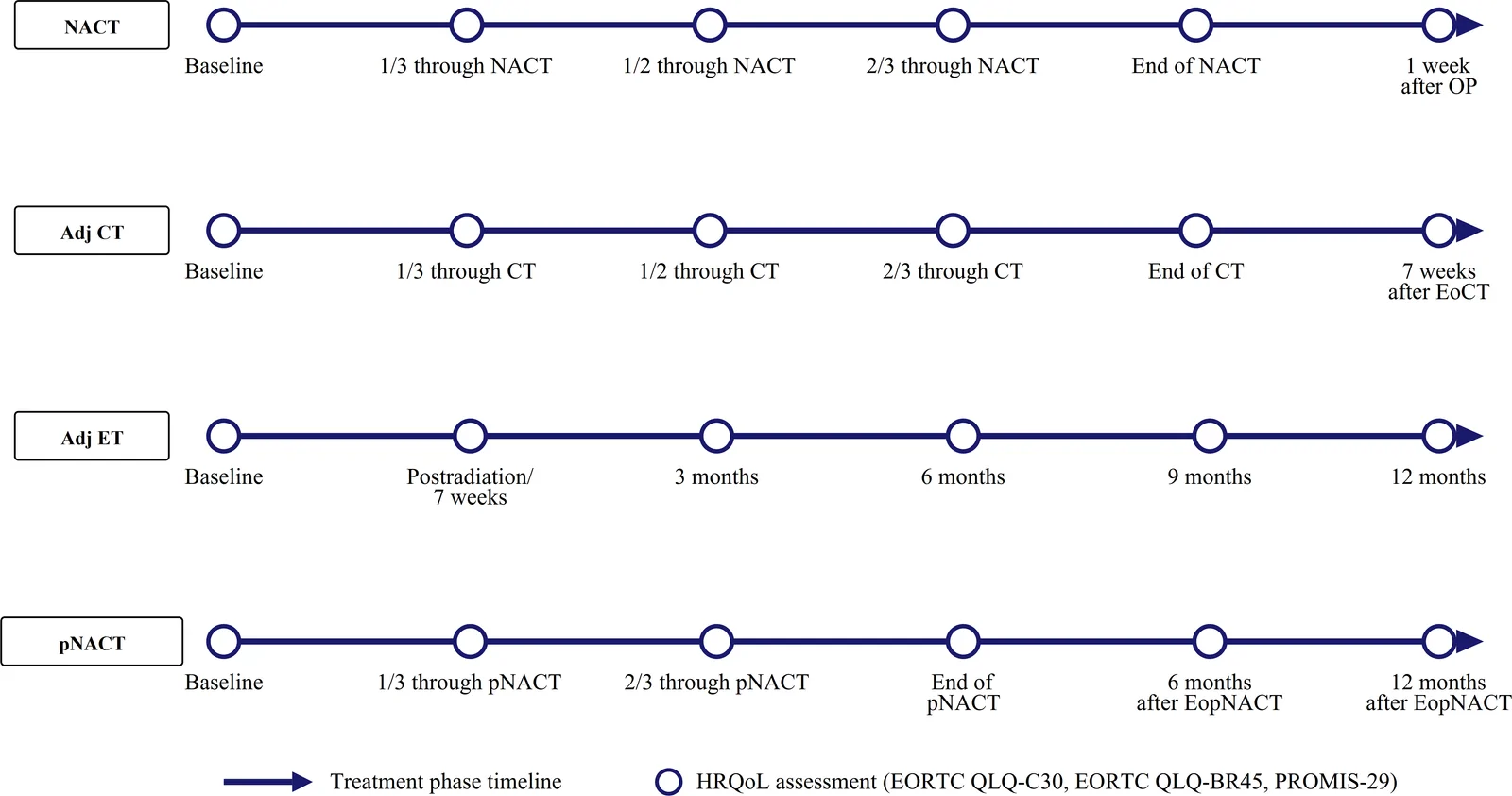
Time points of HRQoL assessments across the 4 therapeutic pathways in the ENABLE trial. ENABLE multicenter, open-label randomized controlled trial in adults with early (stage I-III) breast cancer initiating systemic therapy at 2 German university hospitals (Heidelberg and Tübingen). HRQoL was assessed (EORTC QLQ-C30, EORTC QLQ-BR45, and PROMIS-29) at 6 fixed time points; the 3 midtreatment points denote assessments scheduled after one-third, one-half, and two-thirds of the planned adjuvant chemotherapy or NACT course. Each patient followed exactly 1 therapeutic pathway. Each patient was assigned to exactly 1 therapeutic phase. Patients completed assessments digitally through the patient-facing ENABLE native smartphone app (iOS and Android); study teams managed enrollment and clinical workflows through the web-based PIA care-team platform. Adj CT: adjuvant chemotherapy; Adj ET: adjuvant endocrine therapy; CT: chemotherapy; EoCT: end of chemotherapy; EopNACT: end of postneoadjuvant therapy; EORTC QLQ-BR45: European Organisation for Research and Treatment of Cancer Quality of Life Questionnaire Breast 45; EORTC QLQ-C30: European Organisation for Research and Treatment of Cancer Quality of Life Questionnaire Core 30; HRQoL: health-related quality of life; NACT: neoadjuvant chemotherapy; OP: operation (surgery); PIA: ENABLE web-based care-team platform; pNACT: postneoadjuvant therapy; PROMIS-29: Patient-Reported Outcomes Measurement Information System-29.

#### Intervention Arm—ePRO Monitoring With Threshold-Triggered Digital Symptom Escalation (RPA Group)

In addition to the above, participants in the intervention arm completed a brief weekly assessment of current HRQoL via the app using the EQ-VAS. If a clinically meaningful decline was detected—defined as an EQ-VAS score below 50, or a decrease of ≥10-points relative to the previous score—a rule-based digital triage and escalation pathway with care-team integration was automatically triggered (Figure 1):

1. Symptom assessment: A digital questionnaire prompted patients to report therapy-related adverse events, graded by severity according to an ePRO adaptation of the Common Terminology Criteria for Adverse Events (CTCAE). The queried symptom domains are included in Table S1 in Multimedia Appendix 1. Each item used an ordinal severity scale with a maximum grade of 3 (eg, 0=none to 3=worst possible or functionally limiting); the adapted ePRO instrument did not include CTCAE grades 4‐5, so a grade of 3 denotes the highest self-reported severity.
2. Psycho-oncological screening: A graduated psychological assessment was conducted using the Patient Health Questionnaire-8, the Generalized Anxiety Disorder 7-item scale, and the European Organisation for Research and Treatment of Cancer Quality of Life Questionnaire Fatigue 12 (EORTC QLQ-FA12) fatigue module.
3. Patient-facing response: Patients received evidence-based educational content on managing the reported adverse events via the app. For symptoms graded CTCAE grade 2 or 3, patients were additionally prompted to contact their treatment team directly.
4. Care-team integration and response: In parallel, the care team was notified in real time via the care-team platform as a dashboard flag. In case of an ePRO-adapted CTCAE symptom severity of grade 3 (the maximum of the adapted scale), the care team contacted the patient by telephone or email within 24 hours.

This combined process—weekly EQ-VAS, rule-based digital triage, and escalation pathway with care team integration—is referred to as RPA.

#### Control Arm—ePRO Monitoring Alone (Control Group)

Participants in the control arm completed the same 6 scheduled HRQoL assessments and had access to the same app-based educational and organizational content. They did not receive the weekly EQ-VAS screening, the automated symptom triage, or the real-time care-team alerts. ePRO data collected in the control arm were visualized on the care-team platform but did not trigger automated notifications or escalation workflows. The control condition therefore represented enhanced digital support beyond usual care rather than care-as-usual: both arms received app-based ePRO monitoring and educational content, and control-arm ePRO data were available to the care team for routine review but were not algorithmically triaged or escalated. This design isolates the incremental effect of real-time, threshold-triggered triage and care-team escalation rather than the effect of ePRO collection alone.

App use was supported by predefined email reminders. Reminder intervals were set according to questionnaire type: 3 days after issuance for EQ-VAS assessments (intervention arm) and 7 days after issuance for all other ePRO assessments (both arms). This reminder logic was prospectively specified, technically implemented, and verified through internal testing procedures before trial deployment.

Conceptually, the intervention operationalized a patient-centered approach to supportive oncology care rather than a single named behavior change theory. The intervention was developed within an iterative development framework including stakeholder-informed design processes and structured usability testing. Elements of cocreation workshops and standardized usability assessment informed refinement of the platform prior to clinical deployment. Detailed usability outcomes have been reported separately within the ENABLE development framework [26]. Clinical content, escalation thresholds, and patient-facing intervention logic were defined through expert consensus within the interdisciplinary study team and implemented as rule-based algorithms. Accuracy and functionality were verified through internal testing and validation procedures prior to study initiation.

No major software bugs, content revisions, or intervention-relevant downtimes affecting trial delivery were identified during the study period. Platform operation remained stable for routine clinical use.

### Outcomes

The ethics-approved protocol named change in HRQoL and treatment satisfaction, measured with PROMIS-29, in its statement of the primary end point; the trial registration (DRKS00025611) names the same 2 constructs without specifying an instrument. The protocol did not, however, provide a confirmatory framework for treatment satisfaction: the only instrument named in that statement was the PROMIS-29, which contains no treatment-satisfaction scale; the protocol’s secondary end points included the between-group comparison of patient satisfaction using the PSQ-18; the sample-size calculation rested on HRQoL measured with PROMIS-29 alone; and the protocol directed that secondary end points be analyzed descriptively and interpreted without confirmatory implication. The primary end point is therefore reported here as change in HRQoL measured with PROMIS-29. Because the protocol did not designate a single PROMIS-29 domain as primary, define a composite PROMIS-29 score, or specify a binary global success rule, the primary HRQoL end point is reported as an 8-measure family comprising the 7 PROMIS-29 domains plus the pain-intensity item. Patient satisfaction was evaluated through a single administration of the PSQ-18 at the conclusion of therapy and is reported as a secondary end point, in line with the protocol’s list of secondary end points and with what it specified operationally. This divergence from the wording of the primary end-point statement and of the registry entry is disclosed here, and no statistical analyses were changed by this clarification.

The PROMIS-29 is a standardized, self-report questionnaire designed to measure key dimensions of HRQoL across various chronic conditions and populations. It includes 7 domains: physical function, anxiety, depression, fatigue, sleep disturbance, ability to participate in social roles and activities, and pain interference. They are assessed by 4 items each, plus 1 item for pain intensity. Domain scores were expressed as *T*-scores on the standard Patient-Reported Outcomes Measurement Information System metric (mean 50, SD 10; calibrated to the US general population). The EORTC QLQ-C30 and EORTC QLQ-BR45 were administered at the same 6 time points as prespecified secondary HRQoL measures; their full analysis is reported separately, and here, they serve only as an internal directional concordance check against the PROMIS-29 primary end point.

Patient satisfaction was assessed using the self-assessed PSQ-18, an 18-item measure derived from the PSQ-III (RAND Corporation), yielding 7 subscale scores and a global satisfaction score [27]. To better reflect the German health care context and the study’s focus on adverse event management, the Financial Aspects subscale was replaced with a Management of Adverse Events subscale (2 adapted items; details in the study protocol [24]).

### Statistical Methods

The target sample size was 400 (200 per group), derived for the primary end point (change in HRQoL by PROMIS-29), assuming a minimally important difference (MID) of 5 points on the PROMIS *T*-score metric (SD 10), that is, half an SD, 2-sided α=.05, and 80% power for a repeated-measures (mixed) analysis; the base requirement of n=250 (125 per arm) was inflated by 37.5% to account for anticipated missingness or attrition, yielding the final target n=400. Calculations followed the protocol’s statistical design for the interventional subprotocol. Following a favorable recruitment trajectory, the target was increased to n=600 (ethics committee amendment S-658/2020, approved 27 October 2022); this increase was independent of the interim analysis of the secondary end points and was not informed by its results. The protocol did not designate a single visit as the primary time point; the primary analysis is the longitudinal group×visit comparison across the 6 assessments, with end-of-treatment contrasts interpreted against the 5-point MID.

### Analysis Population

#### Overview

The main comparative analysis of the primary end point used an mITT approach, which included all randomized participants who provided at least 1 postrandomization assessment of the primary end point [28,29]. The baseline assessment was completed in the study portal after central randomization and therefore counts toward this criterion. In accordance with ICH E9 (International Council for Harmonisation of Technical Requirements for Pharmaceuticals for Human Use Efficiency Guideline 9) standards, participants who provided no assessment were excluded from the mITT analysis, as they contributed no data for evaluating treatment effects [30].

#### Primary Analysis: PROMIS-29 Trajectory Models

##### Overview

Primary longitudinal outcomes were analyzed for each of the 8 PROMIS-29 measures using linear mixed-effects models with fixed effects for treatment group (RPA vs control), visit (categorical; 6 levels including baseline), and their interaction, and a random intercept per participant: y(measure)~group×visit+(1|PatientID). Regression coefficients with Wald 95% CIs and model-based marginal means are reported. To test whether trajectories differed by group, the full model was compared to a reduced model without the interaction using likelihood-ratio tests (*df*=5). The marginal *R*^2^ (Nakagawa and Schielzeth framework) quantified variance explained by fixed effects. Analyses assumed data missing at random and used all available observations without imputation; no baseline covariates were included. As an exploratory check of effect consistency across treatment settings, a treatment group×therapy-pathway interaction (neoadjuvant or adjuvant chemotherapy, adjuvant endocrine, and postneoadjuvant) was added to each PROMIS-29 trajectory model and tested by likelihood-ratio test.

##### Multiplicity Control and Interpretation

The primary analysis tested the group-by-visit interaction across the 8 PROMIS-29 measures. *P* values from the corresponding likelihood-ratio tests were adjusted using the BH procedure to control the false discovery rate across the 8 measures. This multiplicity adjustment was applied at the analysis stage and is reported transparently here; it was not described as prespecified in the ethics-approved protocol. Because no single primary domain, composite PROMIS-29 score, or binary global success rule was prespecified, the trial is interpreted by reporting which individual PROMIS-29 measures showed trajectory differences after multiplicity adjustment and by interpreting end-of-treatment contrasts against the 5-point MID. Time-to-deterioration (TTD) analyses for the PROMIS-29 domains, all PSQ-18 comparisons, and subgroup analyses are reported as exploratory without multiplicity adjustment and should be interpreted accordingly.

##### Missing Data

Missingness was characterized separately at the item level (skipped items within a started assessment) and the participant level (visit-wise attrition), and the assumption of data missing completely at random was tested with the Little test. As a sensitivity analysis for the primary trajectory models, missing PROMIS-29 values were multiply imputed (*mice* package, *m*=10 imputations), and the visit-6 between-group contrasts were re-estimated and pooled across imputations.

### Exploratory Analysis: TTD

For each PROMIS-29 domain, Cox proportional-hazards models were fitted with treatment group (RPA vs control) as the sole covariate. Time origin was baseline; follow-up was restricted to the first 6 scheduled visits. Deterioration was defined as a ≥5-point change in the domain *T*-score consistent with worse health (+5 for symptom domains and −5 for functioning domains), in line with established MIDs [31]. Patients were included if they had a valid baseline and ≥1 postbaseline measurement, and deterioration was possible at baseline (ie, not at ceiling or floor). Participants without an event were censored at last assessment; hazard ratio (HR) <1 favors RPA. To assess robustness to the choice of MID, this analysis was repeated using alternative deterioration thresholds of 3, 5, 7, and 10 *T*-score points.

### Secondary Analysis: Patient Satisfaction (PSQ-18)

#### Overview

The PSQ-18 was scored per the instrument manual, with negatively worded items reverse-coded so that higher values indicate greater satisfaction. Seven subscale scores and a global score were computed as item means; incomplete subscales were set to missing without imputation. Between-group differences were assessed using Wilcoxon rank sum tests; *P* values are exploratory without multiplicity adjustment.

All analyses were conducted using R (version 4.5.2; R Foundation for Statistical Computing). The data monitoring committee consisted of the principal investigators from the Heidelberg and Tübingen centers and operated independently of the study sponsor. In accordance with the protocol, a single interim analysis restricted to the secondary end points was performed after approximately 50% of the planned sample had been enrolled; no formal stopping rules or interim efficacy or futility boundaries were specified in the protocol, and the trial was not stopped early. The confirmatory primary HRQoL (PROMIS-29) analysis was not subject to interim testing or α-spending.

#### Statistical Software

All analyses used key R packages including *lme4* (linear mixed-effects models), *emmeans* (marginal means), *performance* (marginal *R*^2^), *survival* (Cox models), *ggsurvfit* (Kaplan-Meier plots), and *stats* (BH adjustment). For a detailed list of used packages, see Table S2 in Multimedia Appendix 1.

### Completion of Scheduled Outcome Assessments

Completion of the scheduled outcome questionnaires was analyzed separately from intervention engagement. This analysis concerned the 6 fixed HRQoL assessment visits completed by participants in both trial arms and was not intended to measure adoption of the RPA intervention itself. Complete discontinuation of scheduled outcome assessment was defined as the first missed visit from which all subsequent scheduled questionnaires remained unanswered. Patients who continued to complete at least 1 scheduled questionnaire until the end of follow-up were right-censored at their last observation. Within the intervention arm, delivered intervention dose was summarized separately: the number of patients triggering at least 1 reactive symptom alert, alerts per patient, the proportion of acknowledged alerts followed by documented care-team contact, and adherence to the weekly EQ-VAS self-assessment.

## Results

### Recruitment

Recruitment ran from March 22, 2021, to September 8, 2023, ending after the amended target sample size of 600 had been reached (605 patients were centrally randomized); the last study visit occurred in February 2024.

### Participant Flow

In total, 1066 patients were assessed for eligibility (Figure 3). Of these, 461 were excluded: 302 did not meet inclusion criteria, 72 declined participation, and 87 were eligible but enrolled in a competing trial. The remaining 605 patients were centrally randomized. A total of 4 participants were retrospectively excluded after randomization due to newly identified metastatic disease, yielding 601 patients in the relevant allocation set (Figure 3). A total of 57 participants discontinued after randomization (withdrawal of consent or no activation of the study app; RPA: n=25; control: n=32). A further 57 participants had no postrandomization HRQoL assessment (RPA: n=33; control: n=24) and are shown in Figure 3 as lost to follow-up for the primary outcome. The mITT population for HRQoL analyses comprised 487 participants (RPA: n=242; control: n=245).

**Figure 3.**
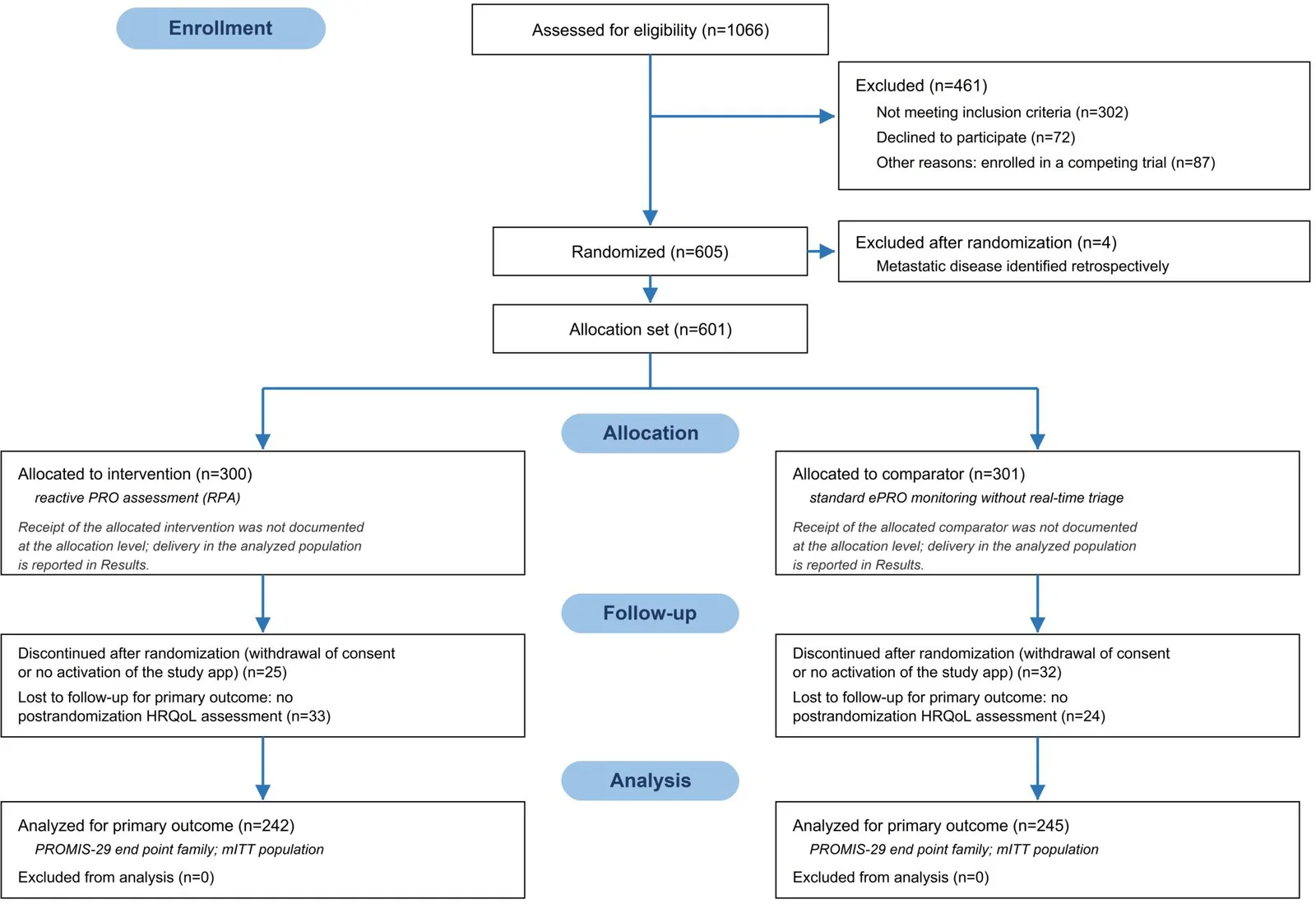
CONSORT 2025 flow diagram of participants in the ENABLE trial. Multicenter, open-label randomized controlled trial in adults with early (stage I-III) breast cancer initiating systemic therapy at 2 German university hospitals (Heidelberg and Tübingen); recruitment March 22, 2021-September 8, 2023. Participants were centrally randomized 1:1 to RPA versus control; the mITT population comprised all randomized participants with at least 1 postrandomization HRQoL assessment. The diagram follows the CONSORT 2025 4-phase template (enrollment, allocation, follow-up, and analysis). Of 1066 patients assessed for eligibility, 461 were excluded (302 not meeting inclusion criteria, 72 declined, and 87 enrolled in a competing trial), and 605 were centrally randomized; 4 were excluded after randomization for retrospectively identified metastatic disease, leaving 601 in the allocation set (RPA: n=300; control: n=301). Participants with no postrandomization HRQoL assessment (RPA: n=33; control: n=24) are shown as lost to follow-up for the primary outcome, consistent with the CONSORT 2025 template. The analysis boxes therefore show the primary-outcome mITT population (RPA: n=242; control: n=245) without a second exclusion of these participants at the analysis stage. CONSORT: Consolidated Standards of Reporting Trials; ePRO: electronic patient-reported outcome; HRQoL: health-related quality of life; mITT: modified intention-to-treat; PRO: patient-reported outcome; PROMIS-29: Patient-Reported Outcomes Measurement Information System-29; RPA: reactive patient-reported outcome assessment.

### Completion of Scheduled Outcome Questionnaires

Completion of scheduled outcome questionnaires was examined as a process measure across the 6 fixed HRQoL assessment visits. Time to complete discontinuation of scheduled outcome assessment was analyzed using Kaplan-Meier curves and univariable Cox models. Continued completion of scheduled questionnaires was similar between trial arms, with no evidence of a between-group difference in time to discontinuation (HR 1.06, 95% CI 0.75‐1.48; *P*=.75; Figure S1 in Multimedia Appendix 1). At 26 weeks, corresponding to the median treatment duration in the sample, estimated continued completion was 78.2% in the control group and 79.8% in the RPA group. These estimates refer to scheduled outcome-questionnaire completion in both groups and are distinct from weekly EQ-VAS adherence and reactive-alert engagement in the intervention arm.

### Baseline Data

Among the 487 patients included in the mITT population, 329 (67.6%) were scheduled to receive neoadjuvant chemotherapy at the time of enrollment, 115 (23.6%) adjuvant chemotherapy, 33 (6.8%) adjuvant endocrine therapy, and 10 (2.1%) postneoadjuvant therapy. Patient characteristics for the control and intervention groups are detailed in Table 1.

No relevant differences in age, therapy situation, type of systemic therapy, Eastern Cooperative Oncology Group status at enrollment, trial site, tumor characteristics, or other important confounders were observed between the 2 study groups following randomization.

**Table 1.** Baseline characteristics of study participants by intervention arm (control vs reactive patient-reported outcome assessment [RPA]) within modified intention-to-treat (mITT) analysis^a^.

Characteristics	Overall (n=487)	Treatment received	
		Control (n=245)	RPA (n=242)
Trial site, n (%)			
Heidelberg	291 (60)	148 (60)	143 (59)
Tübingen	196 (40)	97 (40)	99 (41)
Age			
Mean (SD) (years)	50 (10)	50 (11)	51 (10)
Range (years)	27-82	27-82	27-77
Median (IQR) (years)	51 (42-58)	50 (42-57)	51 (43-58)
Missing or not applicable, n	46	24	22
ECOG^b^ or WHO^c^ status at enrollment, n (%)			
0	253 (94)	124 (96)	129 (93)
1	14 (5.2)	5 (3.9)	9 (6.5)
2‐4	1 (0.4)	0 (0)	1 (0.7)
Missing or not applicable	219	116	103
Menopausal status at enrollment, n (%)			
Premenopausal	242 (52)	119 (52)	123 (52)
Postmenopausal	193 (41)	96 (42)	97 (41)
Perimenopausal	32 (6.9)	15 (6.5)	17 (7.2)
Missing or not applicable	20	15	5
Clinical T-stage of current disease at diagnosis, n (%)			
T1	143 (29)	76 (31)	67 (28)
T2	256 (53)	120 (49)	136 (56)
T3 or T4	66 (14)	35 (14)	31 (13)
cTis or other	22 (4.5)	14 (5.7)	8 (3.3)
Clinical N-stage of current disease at diagnosis, n (%)			
N0	299 (61)	159 (65)	140 (58)
N+	177 (36)	81 (33)	96 (40)
NX	11 (2.3)	5 (2)	6 (2.5)
Grading of current disease at diagnosis, n (%)			
G1	9 (1.9)	3 (1.2)	6 (2.6)
G2	223 (47)	119 (49)	104 (45)
G3	242 (51)	119 (49)	123 (53)
Missing or not applicable	13	4	9
Estrogen receptor status at diagnosis, n (%)			
Positive	308 (64)	155 (65)	153 (64)
Negative	171 (36)	85 (35)	86 (36)
Missing or not applicable	8	5	3
Progesterone receptor at diagnosis, n (%)			
Positive	263 (55)	133 (55)	130 (55)
Negative	214 (45)	107 (45)	107 (45)
Missing or not applicable	10	5	5
Human epidermal growth factor receptor 2 receptor status at diagnosis, n (%)			
Positive	161 (34)	84 (35)	77 (33)
Negative	313 (66)	154 (65)	159 (67)
Missing or not applicable	13	7	6
Ki-67 proliferation index at diagnosis			
Mean (SD)	45 (24)	44 (24)	46 (24)
Range	1-100	5-100	1-95
Median (IQR)	40 (25-60)	40 (25-60)	40 (25-65)
Missing or not applicable	18	9	9
Side of operation, n (%)			
Left	84 (52)	44 (54)	40 (51)
Right	68 (42)	33 (40)	35 (44)
Both sides	9 (5.6)	5 (6.1)	4 (5.1)
Missing or not applicable	326	163	163
Received therapy, n (%)			
Neoadjuvant chemotherapy	329 (68)	163 (67)	166 (69)
Adjuvant chemotherapy	115 (24)	63 (26)	52 (21)
Adjuvant endocrine therapy	33 (6.8)	16 (6.5)	17 (7.0)
Postneoadjuvant therapy	10 (2.1)	3 (1.2)	7 (2.9)

a ENABLE multicenter, open-label randomized controlled trial in adults with early (stage I-III) breast cancer initiating systemic therapy (Heidelberg and Tübingen, Germany; recruitment 2021‐2023); modified intention-to-treat population (n=487; intervention [RPA] vs control). Population: mITT (operational definition in the Methods section); groups: columns reflect randomized assignment (control vs RPA) within the mITT population; denominators: percentages are calculated among nonmissing observations within each column; missingness: “Missing or not applicable” indicates unavailable values or variables not applicable to a given patient; denominators vary accordingly. Timing: baseline clinical characteristics are recorded at diagnosis or enrollment before initiation of systemic therapy unless stated otherwise. Staging or grades: clinical T and N stage at diagnosis; tumor grade G1-G3 per local pathology; receptors/Ki-67: estrogen receptor/progesterone receptor/human epidermal growth factor receptor 2 status at diagnosis; Ki-67 reported as percentage of positively staining tumor cells; surgery or radiation fields:

b ECOG: Eastern Cooperative Oncology Group.

c WHO: World Health Organization.

### Intervention and Comparator Delivery

#### Overview

In the control arm, participants received the same patient-facing native smartphone app, educational and organizational content, reminders, and 6 scheduled HRQoL assessments, but did not receive weekly EQ-VAS screening, automated symptom triage, care-team alerts, or reactive contact triggered by the RPA pathway. In the intervention arm of the mITT population, 209 of 242 patients had monitoring data available for analysis; a further 33 did not start therapy or had no follow-up data. Of these 209 monitored patients, 160 (76.6%) triggered at least 1 reactive symptom alert (median 2 per patient, IQR 1‐3; 508 alert episodes in total). Documented care-team contact followed 339 of 583 (58.1%) acknowledged alerts, and 138 patients had at least 1 such contact. Adherence to the weekly EQ-VAS self-assessment was a median of 66% per patient (IQR 39-91; overall 4819/8131 expected weekly assessments, 59.3%). Structured categories of clinical actions after such contacts were not available in the analysis dataset; symptom-level alert characteristics and response processes are the subject of a dedicated analysis of the ENABLE reactive-monitoring data.

#### Missing Data

Item-level missingness among started assessments was negligible (154/65, 184 items, 0.24%). Missing data were predominantly monotone participant-level attrition: the proportion of participants assessed declined from 408 of 487 (83.8%) at visit 1 to 323 of 487 (66.3%) at visit 6 and was balanced across arms (visit 6: RPA: 158/242, 65.3%; 95% CI 59.1‐71.0; control: 165/245, 67.3%; 95% CI 61.2‐72.9). The Little test rejected the assumption of data missing completely at random (*χ*^2^_1390_=1771.0; *P*<.001), consistent with a missing-at-random mechanism under which the mixed-effects models using all available observations remain valid. In a multiple-imputation sensitivity analysis (*m*=10), the visit 6 effects on fatigue (−3.65; *P*<.001) and social participation (+2.74; *P*=.001) remained robust, whereas the anxiety effect attenuated to borderline significance (−1.64; *P*=.08).

### Numbers Analyzed, Outcomes, and Estimation

Table 2 presents the global likelihood-ratio tests derived from the linear mixed-effects models for the 8 PROMIS-29 measures. These models address whether the RPA intervention altered the longitudinal trajectory of each PROMIS-29 measure across the 6 scheduled assessments. After BH adjustment across the 8-measure family, statistically significant trajectory differences were observed for anxiety, fatigue, and social participation. Physical function, depression, sleep disturbance, pain interference, and pain intensity did not show adjusted trajectory differences. Because no binary global success rule was prespecified for the family, these findings are reported as domain-specific trajectory differences rather than as a single global statement that the primary end point was achieved.

**Table 2.** Global likelihood-ratio (LR) tests for interaction effects between group allocation and measurement time points (“Does RPA alter the trajectory of HRQoL throughout the entire course of therapy?”)^a^.

PROMIS-29^b^ subscale	LR test (*df*=5)	*P* value (crude)	Adjusted *P* value (Benjamini-Hochberg)	Values, n^c^
Physical function	7.9	.16	.18	487
Anxiety	13.6	.02	.049	486
Depression	10.4	.07	.10	486
Fatigue	21.9	<.001	.004	486
Sleep disturbance	12.8	.03	.051	486
Social participation	18.2	.003	.01	486
Pain interference	8.2	.15	.18	486
Pain intensity	5.9	.32	.32	486

a Primary health-related quality of life (HRQoL) analysis, ENABLE multicenter open-label randomized controlled trial in early (stage I-III) breast cancer during systemic therapy (Heidelberg and Tübingen, Germany; 2021‐2023); modified intention-to-treat population (n=487; intervention [reactive patient-reported outcome assessment] vs control). Test: global LR tests of the group×visit interaction vs a model without the interaction; 2-sided *P* values reported; degrees of freedom (5): with group (2 levels) and visit (6 levels), the interaction adds (2−1)×(6−1)=5 parameters in lmer(*y*~group*visit+(1|PatientID)) vs lmer(*y*~group+visit+(1|PatientID)); LR *df* equals the number of added parameters; multiplicity: Benjamini-Hochberg adjustment across the 8 PROMIS domains (reported as “adjusted *P* value”).

b PROMIS-29: Patient-Reported Outcomes Measurement Information System-29.

c Participants contributing ≥1 postbaseline measurement to the mixed-effects model.

Figure 4 presents the estimated marginal means from the post-hoc linear mixed-effects models for each PROMIS-29 subscale over all study visits (regression coefficients in Table S3 in Multimedia Appendix 1). By the sixth visit, the RPA group showed more favorable trajectories for 5 of 8 domains, with the largest effects observed for fatigue (*b*=–3.90, 95% CI –5.77 to –2.03) and social participation (*b*=2.84, 95% CI 1.18‐4.49). No relevant group differences were observed for pain interference and pain intensity. The marginal *R*^2^ ranged from 0.8% (sleep disturbance and pain intensity) to 6.5% (physical function). Although statistically significant, the between-group differences at the final visit for all 3 affected domains remained below the prespecified 5-point MID—anxiety −2.10 (95% CI −3.65 to −0.55), fatigue −3.90 (95% CI −5.77 to −2.03), and social participation 2.84 (95% CI 1.18 to 4.49; Table S3 in Multimedia Appendix 1)—indicating statistically detectable but subthreshold effects on these domains. The treatment effect was consistent across therapy pathways: no group×therapy-pathway interaction reached statistical significance for any PROMIS-29 domain (all *P*≥.22), although power to detect interaction was limited in the small adjuvant-endocrine and postneoadjuvant subgroups.

**Figure 4.**
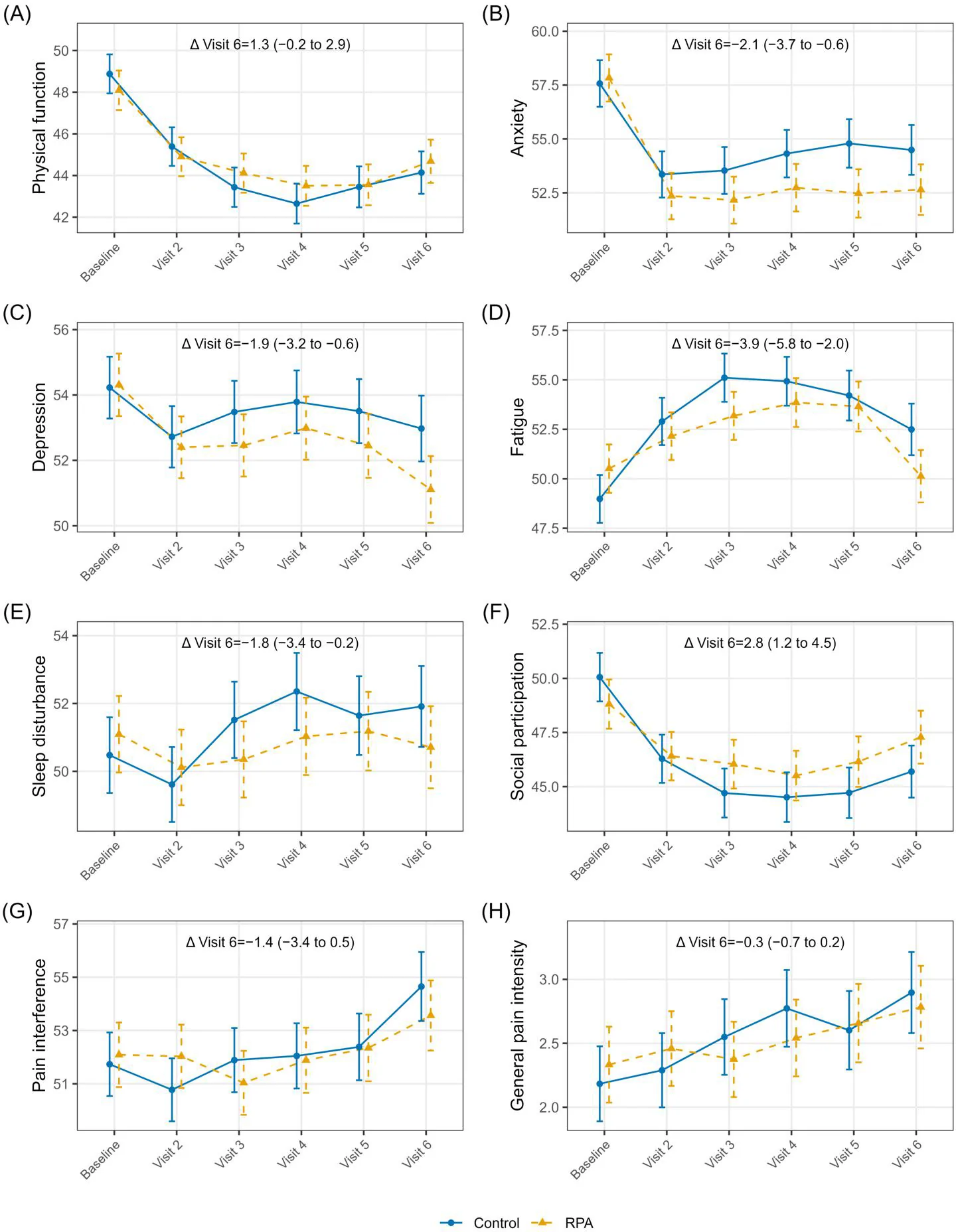
Estimated marginal means (±95% CIs) for each PROMIS-29 subscale (*T*-scores) across study visits, derived from the linear mixed-effects models; each panel is annotated with the end-of-treatment (visit 6) between-group difference and its 95% CI (see Table 2 for likelihood-ratio tests and Table S3 in Multimedia Appendix 1 for regression coefficients). ENABLE multicenter open-label randomized controlled trial in early (stage I-III) breast cancer during systemic therapy (Heidelberg and Tübingen, Germany; 2021‐2023); modified intention-to-treat population (n=487; intervention [RPA] vs control). Shown are trajectories from baseline to visit 6 for both the RPA and control groups. The plots illustrate differences in the course of health-related quality of life domains over time between study groups. (A) Physical function, (B) anxiety, (C) depression, (D) fatigue, (E) sleep disturbance, (F) social participation, (G) pain interference, and (H) general pain intensity in the past 7 days. In each panel, “Δ Visit 6” denotes the between-group difference in change from baseline to the end of treatment (the group×visit interaction at visit 6; RPA-control) with its 95% CI; for the *T*-score domains, this is interpretable against the 5-point minimally important difference. Error bars are 95% CIs; analyzed n=245/242 (control/RPA) for physical function and 244/242 for the remaining domains. PROMIS-29: Patient-Reported Outcomes Measurement Information System-29; RPA: reactive patient-reported outcome assessment.

### Ancillary Analyses

#### Exploratory TTD Analysis

These findings are further supported by TTD analyses. As shown in Table S4 in Multimedia Appendix 1, Cox proportional hazards models indicated a lower risk of clinically meaningful deterioration—defined as an increase or decrease of 5 *T*-score points—in the RPA group for several PROMIS-29 domains. Specifically, participants in the RPA group exhibited a reduced hazard of worsening in depression (HR 0.66, 95% CI 0.44‐0.98), fatigue (HR 0.69, 95% CI 0.54‐0.89), and social participation (HR 0.76, 95% CI 0.59‐0.97) compared to the control group. These findings are based on exploratory *P* values <.05 and should be interpreted accordingly. The corresponding Kaplan-Meier curves are shown in Figure 5. Notable trends favoring RPA are also shown in Table S4 in Multimedia Appendix 1 for physical functioning (HR 0.87, 95% CI 0.67‐1.12), anxiety (HR 0.69, 95% CI 0.45‐1.07), and sleep disturbance (HR 0.85, 95% CI 0.64‐1.12). In a sensitivity analysis using alternative deterioration thresholds (3, 5, 7, and 10 *T*-score points), the reduced risk of deterioration seen at the 5-point threshold was also present at the alternative thresholds for fatigue (3 points: HR 0.74, 95% CI 0.59-0.94; 7 points: HR 0.70, 95% CI 0.54-0.91; 10 points: HR 0.71, 95% CI 0.52-0.96) and at the 7- and 10-point thresholds for social participation (7 points: HR 0.72, 95% CI 0.54-0.95; 10 points: HR 0.72, 95% CI 0.52-0.98; borderline at the 3-point threshold: HR 0.81, 95% CI 0.64-1.02; *P*=.07), whereas the depression effect reached statistical significance only at the 5-point threshold, indicating greater sensitivity to the chosen MID.

**Figure 5.**
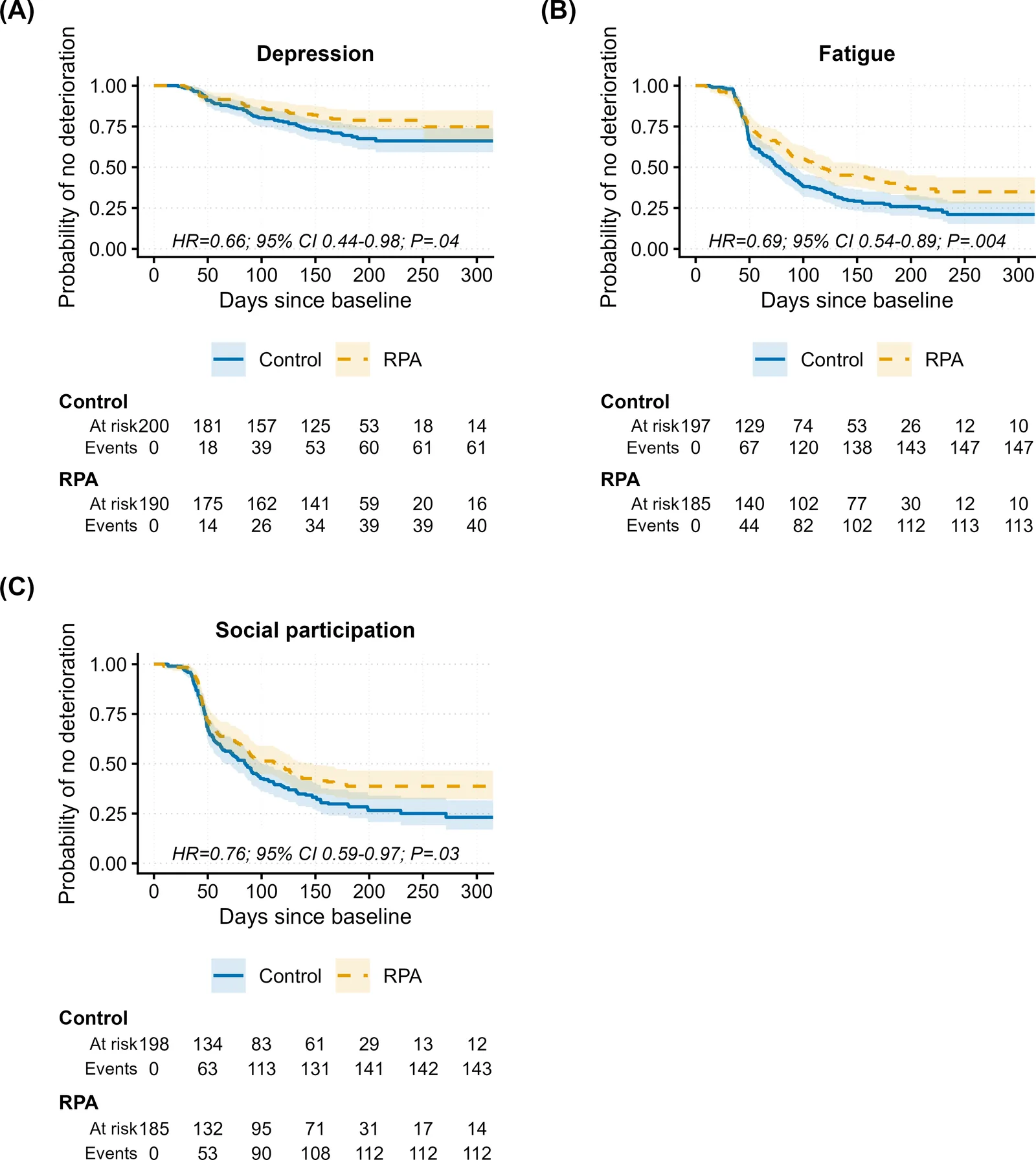
Time to deterioration for PROMIS-29 depression, fatigue, and social participation. Exploratory time-to-deterioration analysis, ENABLE multicenter open-label randomized controlled trial in early (stage I-III) breast cancer during systemic therapy (Heidelberg and Tübingen, Germany; 2021‐2023); modified intention-to-treat population (n=487; intervention [RPA] vs control). Kaplan-Meier curves depict the time from therapy initiation to the first clinically meaningful deterioration (≥5-point change in *T*-score) for each outcome; shaded areas represent 95% CIs. (A) Depression, (B) fatigue, and (C) social participation. HR: hazard ratio; PROMIS-29: Patient-Reported Outcomes Measurement Information System-29; RPA: reactive patient-reported outcome assessment.

#### Patient Satisfaction (PSQ-18)

In all, 329 (67.6%) patients completed the PSQ-18 questionnaire. Comparison of the total score as well as the subscores of the PSQ-18 between the control and intervention groups showed no relevant difference, as illustrated in Figure 6 and Table S5 in Multimedia Appendix 1. Patients receiving neoadjuvant chemotherapy reported higher mean satisfaction scores for “accessibility and convenience,” “time spent with doctor,” “communication,” “technical quality,” “general satisfaction,” and the PSQ-18 total score compared to patients receiving adjuvant chemotherapy. These differences were associated with exploratory *P* values <.05 (Figure S2 and Table S6 in Multimedia Appendix 1). Among patients who received neoadjuvant chemotherapy, no substantial difference in patient satisfaction was observed between those who achieved a pathological complete response and those who did not (Figure S3 and Table S7 in Multimedia Appendix 1).

**Figure 6.**
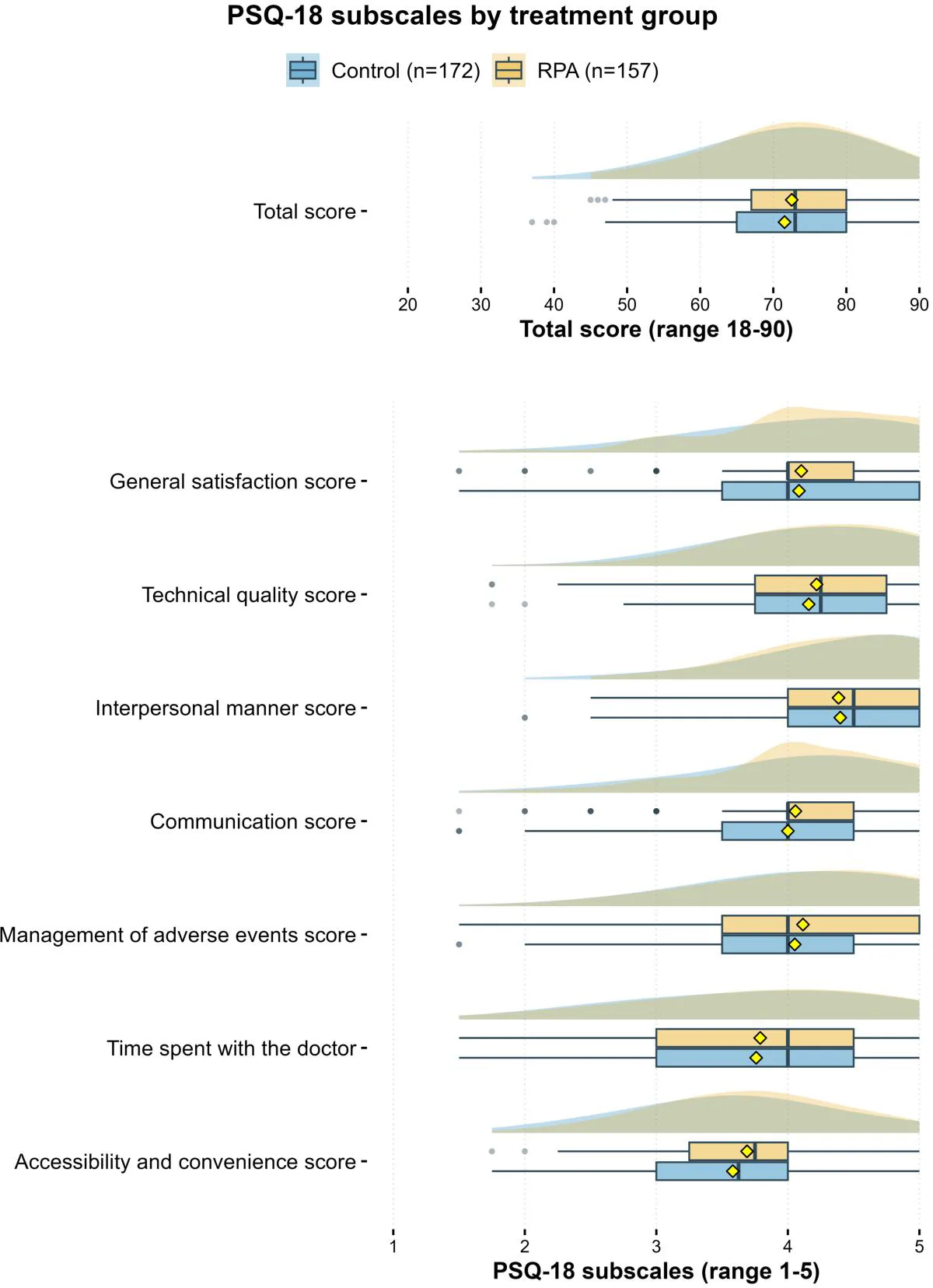
PSQ-18 total and subscale scores comparing RPA intervention (n=157) versus control group (n=172). Patient satisfaction (secondary end point), ENABLE multicenter open-label randomized controlled trial in early (stage I-III) breast cancer during systemic therapy (Heidelberg and Tübingen, Germany; 2021‐2023). Box plots indicate median and quartiles, yellow diamonds mark group means, and violin plots show density distributions in both groups*.* PSQ-18: Patient Satisfaction Questionnaire Short Form; RPA: reactive patient-reported outcome assessment.

### Harms

No intervention-related safety concerns, privacy breaches, or clinically relevant unintended digital effects were identified during the trial. Harms were not ascertained through a predefined adverse-event reporting protocol (see Harms in the Methods section).

## Discussion

### Principal Findings

In this multicenter randomized trial, adding real-time ePRO monitoring with rule-based digital triage and care-team integration to app-based support improved selected PROMIS-29 trajectories compared with an active digital-support comparator. The pattern was domain-specific: anxiety, fatigue, and ability to participate in social roles and activities showed statistically detectable trajectory differences, whereas physical function, sleep disturbance, pain, and depression did not show adjusted trajectory differences in the primary models. All end-of-treatment between-group differences remained below the prespecified PROMIS MID, and treatment satisfaction did not differ between groups. The findings therefore support a targeted and modest benefit for psychological and social well-being rather than a broad or global improvement in HRQoL.

### Comparison With Prior Work

The active comparator is central to interpretation. Unlike trials comparing ePRO monitoring with usual care, both arms in ENABLE received digital app-based information, organizational support, and scheduled HRQoL assessment. This design is closest conceptually to ePRO studies that test a more responsive or interactive intervention against lower-intensity digital support, such as PreCycle, while asking a different question in early breast cancer [32,33]. The modest effect sizes observed here are therefore consistent with an incremental-effect trial: the contrast isolated real-time triage and care-team escalation rather than digital support or ePRO collection alone.

Our findings are broadly consistent with evidence that ePRO monitoring can improve symptom burden, HRQoL, self-efficacy, or supportive-care processes in oncology, including adjuvant-treatment settings [21,34,35]. At the same time, the literature does not support a uniform expectation of large clinical effects across all settings and outcomes. Earlier metastatic-disease studies suggested possible survival benefits of web-based symptom monitoring [13,16], whereas the final Patient-Reported Outcomes to Enhance Cancer Treatment results did not show an overall-survival benefit despite improvements in symptom control, physical function, HRQoL, and emergency-visit outcomes [18]. This mixed evidence supports the cautious framing used here and argues against extrapolating metastatic-disease outcomes directly to a predominantly nonmetastatic early breast cancer cohort.

The domain-specific pattern may reflect the mechanism of the intervention, but this interpretation remains hypothesis-generating. The RPA pathway combined weekly screening, rule-based symptom triage, tailored patient-facing advice, and care-team alerting, features that align with elements associated with stronger ePRO effects in recent syntheses [20]. Such a pathway may be more likely to influence psychological distress, fatigue management, and social participation than pain or physical limitations driven primarily by systemic therapy exposure [3,4]. Because the trial was powered for the PROMIS-29 HRQoL end-point family rather than for domain-specific mechanistic hypotheses, this selective pattern should be confirmed in future studies with explicit end-point hierarchies and mechanism-focused process measures.

The PROMIS-29 and TTD analyses were not identical in what they captured. Fatigue and social participation showed the most consistent pattern across the longitudinal trajectory and exploratory deterioration analyses, whereas anxiety and depression differed by analytic approach. This is plausible because longitudinal mixed models estimate average trajectory differences over scheduled assessments, whereas TTD analyses focus on first clinically meaningful worsening among participants at risk [31,33]. Directional concordance with the prespecified secondary European Organisation for Research and Treatment of Cancer (EORTC) emotional-functioning, social-functioning, and fatigue scales provides some support for the PROMIS-29 pattern, although the full EORTC analyses are reported separately and should not be overinterpreted here.

Treatment satisfaction did not differ between the RPA and control groups. This null finding is plausible because both arms received the ENABLE app and structured digital support, which may have reduced the contrast available for PSQ-18 differences. Trials such as CAPRI (Cancérologie Parcours Région Île-de-France) reported improved patient satisfaction when digital monitoring was compared with usual care, but the comparator and disease context differed from ENABLE [36]. The high overall PSQ-18 scores in this trial are also consistent with prior reports showing generally high satisfaction among patients with oncological disease, which can limit sensitivity to additional intervention effects [37-39].

Exploratory differences in satisfaction between neoadjuvant and adjuvant chemotherapy groups should be interpreted cautiously. These comparisons were not randomized, were not part of the primary end-point hierarchy, and may reflect differences in treatment timing, expectations, tumor biology, perioperative context, and assessment timing. The observation is clinically interesting because neoadjuvant treatment provides an in vivo assessment of tumor response and informs subsequent surgical and systemic decisions [2], but the present trial was not designed to isolate the determinants of satisfaction by therapy pathway. These analyses therefore generate hypotheses for future work rather than evidence for a causal effect of therapy setting on satisfaction.

We are not aware of direct comparisons of treatment satisfaction between neoadjuvant and adjuvant chemotherapy contexts; the available satisfaction literature in oncology addresses age, disease stage, and care setting rather than this contrast [37,38]. The present exploratory findings should therefore be viewed as descriptive. They may nevertheless help explain why intervention effects on satisfaction were difficult to detect: satisfaction appears to be shaped by the broader care relationship and treatment context as well as by digital-support features [8]. Future studies could examine whether ePRO-supported care has different perceived value at distinct points in the early breast cancer pathway, particularly around diagnosis, chemotherapy initiation, surgery, and transition to survivorship. Such work would require a design and end-point hierarchy tailored to patient-experience outcomes rather than post-hoc subgroup comparison.

Taken together, the comparison with prior work supports a bounded conclusion. ENABLE adds evidence that real-time, care team–integrated ePRO triage can produce small benefits in selected HRQoL domains even when the comparator includes digital support and scheduled ePRO assessment. It does not show a broad HRQoL effect, a treatment-satisfaction effect, or effects on clinical or health-service outcomes. This distinction is important for implementation: the intervention should be considered a supportive-care workflow tool with targeted patient-reported benefits, not a stand-alone solution for all treatment-related morbidity.

### Limitations

By design, several limitations should be considered. First, the intervention was compared with an active digital-support comparator rather than usual care, which strengthens the mechanistic question but likely attenuates observable effect sizes. Second, the trial enrolled patients across neoadjuvant chemotherapy, adjuvant chemotherapy, adjuvant endocrine therapy, and postneoadjuvant therapy pathways that differ in duration, clinical context, and assessment timing. Randomization balanced these pathways between arms, and the primary mixed-effects models used visit number as a categorical factor, but the small adjuvant endocrine and postneoadjuvant subgroups limit pathway-specific inference. Third, the trial was designed around patient-reported HRQoL and satisfaction end points; emergency visits, unplanned outpatient visits, hospitalizations, febrile neutropenia, chemotherapy delays, dose reductions, and treatment discontinuations were not collected as comparative outcomes and cannot be inferred from these data.

The exploratory therapy pathway and satisfaction analyses have additional limitations. Patients were not randomized to neoadjuvant versus adjuvant treatment, and these pathways differ by tumor subtype, clinical indication, timing of surgery, and expectations of response [2]. Patients receiving neoadjuvant therapy were assessed in a different clinical context than those receiving adjuvant chemotherapy, and the adjuvant subgroup with available satisfaction data was relatively small. These factors preclude causal interpretation of the observed satisfaction differences between treatment settings.

Generalizability is also bounded. The trial enrolled adults with stage I-III invasive, nonmetastatic breast cancer at 2 German university hospitals, and eligibility required German fluency and access to an internet-enabled device. This likely reflects patients treated in comprehensive academic breast units but may underrepresent community settings, older patients, patients with language barriers, and patients with lower digital access or health literacy. The relatively young cohort and the high proportion receiving neoadjuvant chemotherapy mirror contemporary practice in high-volume centers [2] but may differ from other regions or care systems. Evidence from older oncology populations shows that lack of internet access can strongly limit ePRO feasibility, reinforcing the need to test such workflows in more diverse populations [40].

The retrospective registration is an additional reporting limitation. The first participant was enrolled before public trial registration, although the outcome measures had been specified in the ethics committee submission before recruitment began. The protocol was amended during the trial, most substantially to increase the target sample size in October 2022; these amendments did not affect the outcome measures. The paper discloses this timeline and is accompanied by an Editorial Notice explaining the rationale for the exception. Readers should nevertheless interpret primary outcome and effectiveness claims with the caution appropriate for retrospectively registered randomized trials.

In our cohort, feasibility findings should likewise be interpreted within this selected context. The screening and retention data suggest that app-based ePRO assessment was acceptable for many eligible patients in academic early breast cancer care, and completion of scheduled outcome assessments was similar between groups. However, the analyzed mITT sample reflects participants who provided at least 1 HRQoL assessment, and trial entry required digital access. Although the mITT sample exceeded the original sample size derived for the PROMIS-29 HRQoL analysis, the later amended recruitment target was not fully reflected in the final analyzable cohort. These features support internal interpretation of the randomized comparison while limiting claims about universal implementation readiness.

Future research should move beyond the question of whether ePRO data can be collected and toward how digital triage should be integrated into oncology workflows. Independent prospectively registered trials should specify the end-point hierarchy before enrollment; distinguish patient-reported, implementation, and clinical-service outcomes; and define how care-team responses will be categorized. Mechanism-focused analyses of alert types, contact modes, counseling, referral pathways, and response timing may clarify which components drive benefit. Adaptive prediction models may ultimately help personalize alert thresholds and anticipate deterioration, but such approaches require transparent validation and careful integration into clinical governance [41,42].

### Conclusions

In this trial, the PROMIS-29 was evaluated as an 8-measure end-point family rather than as a single global score. Real-time digital triage and care-team escalation improved trajectories in anxiety, fatigue, and social participation, while the remaining PROMIS-29 measures did not show adjusted trajectory differences. Treatment satisfaction, assessed as a secondary end point, did not differ between groups. The observed effects were modest and below the prespecified MID, indicating a targeted benefit for selected psychological and social HRQoL domains rather than a global improvement; because both arms received app-based support and scheduled ePRO assessment, they speak to the incremental value of real-time triage and care-team escalation. The trial therefore supports the feasibility and potential value of embedding automated symptom-triggered triage into early breast cancer care pathways, especially where the goal is responsive supportive care rather than passive data collection; its value appears most likely where symptom signals can trigger timely, structured, and documented responses from the care team, and ENABLE offers a model that can be adapted and tested in other oncology settings. The results should not be interpreted as evidence for effects on clinical outcomes, health-service use, or broad HRQoL improvement, and the modest sub-MID effects argue for careful implementation and evaluation rather than overstatement. Confirmation should come from independent prospectively registered studies with a clear end-point hierarchy and prespecified implementation and response-process outcomes, which should also determine which patients benefit most, which alert-response pathways are most effective, and how such systems can be integrated equitably into routine care.

## Supplementary material

10.2196/95155 Multimedia Appendix 1 Response formats of the symptom items, R packages used, mixed-effects model coefficients for the Patient-Reported Outcomes Measurement Information System-29 measures, hazard ratios for time to deterioration, Patient Satisfaction Questionnaire Short Form comparisons by trial arm, therapy setting, and pathological complete response, and Kaplan-Meier curves for discontinuation of scheduled questionnaires.

10.2196/95155 Checklist 1 CONSORT 2025 checklist.

10.2196/95155 Checklist 2 CONSORT-eHEALTH checklist (V 1.6.1).

## Abbreviations

BH: Benjamini-Hochberg
CONSORT: Consolidated Standards of Reporting Trials
CONSORT-eHEALTH: Consolidated Standards of Reporting Trials of Electronic and Mobile Health Applications and Online Telehealth
CTCAE: Common Terminology Criteria for Adverse Events
EORTC: European Organisation for Research and Treatment of Cancer
EORTC QLQ-BR45: European Organisation for Research and Treatment of Cancer Quality of Life Questionnaire Breast 45
EORTC QLQ-C30: European Organisation for Research and Treatment of Cancer Quality of Life Questionnaire Core 30
EORTC QLQ-FA12: European Organisation for Research and Treatment of Cancer Quality of Life Questionnaire Fatigue 12
ePRO: electronic patient-reported outcome
EQ-VAS: EuroQol Visual Analogue Scale
GDPR: General Data Protection Regulation
HR: hazard ratio
HRQoL: health-related quality of life
ICH E9: International Council for Harmonisation of Technical Requirements for Pharmaceuticals for Human Use Efficiency Guideline 9
MID: minimally important difference
mITT: modified intention-to-treat
PEF-FB-9: Fragebogen zur Partizipativen Entscheidungsfindung
PRO: patient-reported outcome
PROM: patient-reported outcome measure
PROMIS-29: Patient-Reported Outcomes Measurement Information System-29
PSQ-18: Patient Satisfaction Questionnaire Short Form
RPA: reactive patient-reported outcome assessment
TIDieR: Template for Intervention Description and Replication
TTD: time-to-deterioration
