# Supplementary material for: Real-Time Electronic Patient-Reported Outcome Monitoring With Digital Triage and Care-Team Integration in Early Breast Cancer: ENABLE Randomized Controlled Trial
Source: J Med Internet Res. 2026 Oct 28;28:e95155. doi: 10.2196/95155 (PMC13717432; doi:10.2196/95155)
Supplement: Supplementary file 1 [file jmir-v28-e95155-s002.pdf]

CONSORT 2025 Checklist

Manuscript: Real-Time ePRO Monitoring With Digital Triage and Care-Team Integration in Early Breast Cancer: The ENABLE Randomized Controlled Trial

Journal / Manuscript ID: JMIR (#95155), Revision R2

Trial registration: German Clinical Trials Register DRKS00025611 (registered 6 July 2021; retrospective — see item 2)

The "Reported in" column refers to the manuscript section/heading (page numbers are added at typesetting). Items that do not apply are marked "N/A" with an in-text explanation, as required.

**Section / Topic**	**Item**	**Checklist item**	**Reported in**
Title and abstract			
Title and structured abstract	1a	Identification as a randomised trial in the title	Title
	1b	Structured summary of the trial design, methods, results, and conclusions	Abstract
Open science			
Trial registration	2	Name of trial registry, identifying number (with URL) and date of registration	Abstract (Trial Registration); Methods — Trial registration and protocol availability. Registration was retrospective (registered 6 July 2021; first participant enrolled 22 March 2021); the reason for the delay is stated in Methods — Trial design, and an Editorial Notice is to accompany the article under the journal's exception policy
Protocol and SAP	3	Where the trial protocol and	Methods — Trial registration and protocol availability

		statistical analysis plan can be accessed	(protocol publicly available: JMIR Res Protoc 2025;14:e69855)
Data sharing	4	Where and how the de-identified participant data, statistical code and other materials can be accessed	Data Availability (available from the corresponding author on reasonable request; access restricted under GDPR)
Funding and conflicts of interest	5a	Sources of funding and other support, and role of funders	Funding
	5b	Financial and other conflicts of interest of the manuscript authors	Conflicts of Interest (includes the developer relationship of TMD, AB, MW to the evaluated application)
Introduction			
Background and rationale	6	Scientific background and rationale	Introduction
Objectives	7	Specific objectives related to benefits and harms	Introduction — Objectives
Methods			
Patient and public involvement	8	Details of patient or public involvement	Methods — Patient and public involvement (no patients or members of the public were involved; stated explicitly)
Trial design	9	Description of trial design including type of trial, allocation ratio, and framework	Methods — Trial design (multicentre, open-label, parallel-group, 1:1, superiority)
Changes to trial protocol	10	Important changes to the trial after it commenced, with reason	Methods — Changes to trial protocol (metastatic exclusion; Mannheim non-recruitment); Methods — Sample

			size (amendment 400 → 600, approved 27 Oct 2022); Methods — Outcomes (PSQ-18 reported as secondary; see note below)
Trial setting	11	Settings and locations where the trial was conducted	Methods — Trial setting
Eligibility criteria	12a	Eligibility criteria for participants	Methods — Eligibility criteria
	12b	If applicable, eligibility criteria for sites and for individuals delivering the interventions	Methods — Trial setting (academic breast-cancer centres with an established (neo)adjuvant service; intervention delivered by trained study nurses and treating oncologists)
Intervention and comparator	13	Intervention and comparator with sufficient detail to allow replication (TIDieR)	Methods — Intervention and comparator; Figure 2; Supplementary Table S1
Outcomes	14	Pre-specified primary and secondary outcomes	Methods — Outcomes. Primary: HRQoL, analysed as a family of eight PROMIS-29 measures (seven domains plus the pain-intensity item); P values were adjusted at the analysis stage using Benjamini-Hochberg FDR control across the eight measures. No single domain, no composite score, and no prespecified binary global

			success rule. Secondary: treatment satisfaction (PSQ-18)
Harms	15	How harms were defined and assessed (systematically/non-systematically)	Methods — Harms (non-systematic; no predefined adverse-event protocol; rationale stated)
Sample size	16a	How sample size was determined, including assumptions	Methods — Sample size (n=400 target, MID 5 T-score points, α=.05 two-sided, 80% power, inflated 37.5% for attrition; amended to n=600)
	16b	Explanation of any interim analyses and stopping guidelines	Methods — Sample size (one protocol-specified interim analysis of secondary endpoints at ~50% enrolment; no stopping rules; the primary analysis was not interim-tested)
Randomisation: sequence generation	17a	Who generated the random allocation sequence and the method used	Methods — Randomisation: Sequence generation (generated by the biostatistics unit; R v3.6.3, blockrand v1.5)
	17b	Type of randomisation and details of any restriction (e.g., blocking)	Methods — Randomisation: Sequence generation (site-specific block randomisation, random block sizes 2/4/6/8, 1:1, no stratification or minimisation)
Allocation concealment	18	Mechanism used to implement the	Methods — Allocation

		random allocation sequence	concealment mechanism (central web portal; allocation concealed until assignment)
Implementation	19	Whether enrolling/assigning personnel had access to the allocation sequence	Methods — Implementation (study nurses enrolled participants and triggered central assignment without access to the sequence)
Blinding	20a	Who was blinded after assignment to interventions	Methods — Blinding (open-label; participants, clinical teams, outcome assessors and data analysts unmasked)
	20b	If blinded, how blinding was achieved and similarity of interventions	N/A — open-label trial; owing to the nature of the digital intervention no blinding was performed, and no masking-success assessment was undertaken (stated in Methods — Blinding)
Statistical methods	21a	Statistical methods to compare groups for primary and secondary outcomes, including harms	Methods — Statistical methods
	21b	Definition of who is included in each analysis, and in which group	Methods — Statistical methods (mITT = all randomised participants with ≥1 post-randomisation HRQoL assessment, analysed as randomised)
	21c	How missing data were handled	Methods — Statistical methods (Missing data; MAR;

			multiple-imputation sensitivity analysis, m=10)
	21d	Methods for additional analyses, distinguishing prespecified from post-hoc	Methods — Statistical methods; Results — Ancillary analyses (exploratory analyses labelled as such)
Results			
Participant flow (+ diagram)	22a	For each group, numbers randomly assigned, receiving intended intervention, and analysed for the primary outcome	Results — Participant flow; Figure 3 (CONSORT 2025 flow diagram). PARTIAL: numbers randomly assigned and analysed for the primary outcome are reported for each group; receipt of the allocated intervention was not documented at the allocation level and is therefore stated as such in Figure 3, with delivery in the analysed population reported under Results — Intervention and comparator delivery
	22b	For each group, losses and exclusions after randomisation, with reasons	Results — Participant flow; Figure 3
Recruitment	23a	Dates defining the periods of recruitment and follow-up	Results — Recruitment (22 March 2021 – 8 September 2023; last study visit February 2024)
	23b	If relevant, why the trial ended or was stopped	Results — Recruitment (recruitment ended after the amended

			target sample size of 600 was reached)
Intervention and comparator delivery	24a	Intervention and comparator as actually administered (delivery, adherence, fidelity)	Results — Intervention and comparator delivery
	24b	Concomitant care received during the trial for each group	Methods — Intervention and comparator (app-based components common to both arms)
Baseline data	25	A table of baseline demographic and clinical characteristics for each group	Results — Baseline data; Table 1
Numbers analysed, outcomes and estimation	26	For each primary and secondary outcome, by group: numbers analysed, results, effect size and precision	Results — Numbers analysed, outcomes and estimation; Table 2; Figure 4; Supplementary Table S3
Harms	27	All harms or unintended events in each group	Results — Harms. Harms were not systematically or comparatively assessed (non-pharmacological digital intervention; no predefined adverse-event protocol — see item 15); no intervention-related safety concerns, privacy breaches, or clinically relevant unintended digital effects were identified
Ancillary analyses	28	Any other analyses performed, distinguishing pre-specified from post-hoc	Results — Ancillary analyses (exploratory time-to-deterioration; therapy-pathway interaction; MID

			sensitivity; EORTC concordance; treatment satisfaction)
Discussion			
Interpretation	29	Interpretation consistent with results, balancing benefits and harms	Discussion — Principal Findings; Comparison With Prior Work
Limitations	30	Trial limitations: bias, imprecision, generalisability, multiplicity	Discussion — Limitations

---

Notes on this revision (R2)

Heading names. In line with the editor's request, the manuscript now uses the CONSORT 2025

section/topic names as explicit headings in the Methods and Results. In the Discussion and in the

data-availability statement the headings required by the JMIR *Instructions to Authors* are

retained (Principal Findings; Comparison With Prior Work; Limitations; Conclusions; Data

Availability). CONSORT 2025 provides for this explicitly: *"we do not suggest a rigid structure

for the reporting of randomised trials. Instead, the format of articles should abide by the

journal's individual style and its 'Instructions to Authors.'"* The corresponding items (29, 30, 4)

are mapped above.

Item 10 — outcome reporting. The trial registry lists treatment satisfaction alongside HRQoL

among the primary outcomes. In this report, treatment satisfaction (PSQ-18) is a secondary

endpoint: the sample size calculation and primary longitudinal analysis were

based solely on HRQoL (PROMIS-29), whereas the PSQ-18 was administered once, at the end of therapy,

and could not enter the longitudinal confirmatory framework. This is stated in Methods — Outcomes

and is reported here as a post-commencement change.

Item 22a is PARTIAL, not YES. See the entry above. This is deliberate: marking it YES would overstate what the data support.

What changed against the R1 checklist

	**R1**	**R2**
Manuscript title	wrong — carried the pre-ED-1 title	corrected to the submitted title
Revision	R1	R2
Location pointers	old heading names (Participants; Interventions; Randomization and blinding; Enrolment)	CONSORT topic names per the ED2-4 patch
Item 2	registry details only	+ retrospective registration, reason, Editorial Notice
Item 7	"Introduction (study objectives)"	"Introduction — Objectives" (explicit heading)
Item 14	"primary: HRQoL/PROMIS-29; secondary: PSQ-18"	+ eight-measure endpoint family, BH-FDR, no prespecified success rule
Item 22a	YES	PARTIAL — receipt of allocated intervention not documented at allocation level
Item 27	N/A	now pointed at Results — Harms (the section the ED2-4 patch adds)
Item 10	listed PSQ-18 reclassification in passing	stated explicitly, with the registry discrepancy named

---

Checklist items reproduced from: Hopewell S, Chan AW, Collins GS, Hróbjartsson A, Moher D, Schulz KF, et al. CONSORT 2025 Statement: updated guideline for reporting randomised trials. BMJ. 2025;388:e081123. doi:10.1136/bmj-2024-081123. © 2025 Hopewell et al.; Open Access under CC BY 4.0 (https://creativecommons.org/licenses/by/4.0/).
