# Supplementary material for: Real-Time Electronic Patient-Reported Outcome Monitoring With Digital Triage and Care-Team Integration in Early Breast Cancer: ENABLE Randomized Controlled Trial
Source: J Med Internet Res. 2026 Oct 28;28:e95155. doi: 10.2196/95155 (PMC13717432; doi:10.2196/95155)
Supplement: Supplementary file 2 [file jmir-v28-e95155-s003.pdf]

CONSORT-eHEALTH Checklist (V1.6.1)

Manuscript: Real-Time ePRO Monitoring With Digital Triage and Care-Team Integration in Early Breast Cancer: The ENABLE Randomized Controlled Trial

Journal / Manuscript ID: JMIR (#95155), Revision R2

Trial registration: German Clinical Trials Register DRKS00025611

This checklist reports the eHealth-specific subitems of the CONSORT-EHEALTH extension. The standard CONSORT items (registration, design, randomisation, outcomes, flow, etc.) are reported in the accompanying CONSORT 2025 checklist. "Reported in" refers to the manuscript section/heading.

**Subitem**	**CONSORT-EHEALTH item**	**How addressed**	**Reported in**
1a-i	Developers, sponsors, owners of the application/platform	The ENABLE application and the PIA care-team platform were developed and provided by the Center for Innovative Care and the Institute of Women's Health GmbH (Tübingen, Germany); the study was funded by the Ministry of Science, Research and Arts Baden-Württemberg (ZIV project).	Methods — Intervention and comparator; Funding; Authors' Contributions
1a-ii	Target population / age group	Adults (≥18 years) with histologically confirmed, non-metastatic, invasive, stage I–III breast cancer initiating systemic therapy.	Title; Abstract; Methods — Eligibility criteria
1a-iii	Mode of delivery (web/app/mobile)	The patient-facing ENABLE application was a native smartphone app (iOS/Android). Clinical staff used	Methods — Intervention and comparator; Figure 1 legend

		the separate web-based PIA care-team platform.	
1b	Development history / formative evaluation / usability	Developed through an iterative, stakeholder-informed framework with co-creation workshops and standardized usability testing; detailed usability outcomes are reported in a dedicated companion paper (Anders et al. 2024).	Methods — Intervention and comparator
5 (TIDieR core)	Intervention described in sufficient detail for replication	Reported in line with the TIDieR checklist: components, provider, mode, setting, and schedule.	Methods — Intervention and comparator; Figure 2; Supplementary Table S1
5-viii	Mode of delivery, components/features, theoretical framework	The intervention operationalizes a patient-centered care approach (no single named behavior-change theory). Components: scheduled ePRO assessments, weekly EQ-VAS trigger, rule-based symptom triage (ePRO-adapted CTCAE), psycho-oncological screening (PHQ-8, GAD-7, QLQ-FA12), educational content, and real-time care-team alerting.	Introduction; Methods — Intervention and comparator; Figure 2
5-ix	Usage parameters (dose, optimal timing)	Six fixed HRQoL assessment timepoints across	Methods — Intervention and

		therapy; weekly EQ-VAS in the intervention arm; escalation triggered by EQ-VAS <50 or a ≥10% decline.	comparator; Figure 1
5-x	Level of human involvement (care providers)	The care team was notified in real time via a dashboard flag; at CTCAE grade 3 (maximum of the adapted scale) the team contacted the patient by telephone or e-mail within 24 hours.	Methods — Intervention and comparator (steps 3–4)
5-xi	Prompts / reminders	Predefined e-mail reminders with intervals set by questionnaire type (3 days for EQ-VAS; 7 days for other ePRO assessments).	Methods — Intervention and comparator
5-xii	Co-interventions (training/support)	Both arms received a personalized care plan, a content library, a calendar function, and reminders; the control arm received the same educational content but no triage, alerts, or weekly EQ-VAS.	Methods — Intervention and comparator
6	Outcomes incl. use metrics, adherence/attrition, denominators	Completion of the scheduled outcome assessments (both arms) is reported separately from intervention delivery and engagement (intervention arm only) — these are distinct quantities and are no longer described jointly as "adoption". Technical app-	Methods — Statistical methods (Completion of scheduled outcome assessments); Results — Intervention and comparator delivery; Figure 3; Supplementary Figure S1

		usage metrics (logins, session duration) were not used, with justification. Attrition and denominators are reported by visit; engagement metrics (proportion triggering ≥1 alert, alerts per patient, care-team contacts, EQ-VAS adherence) are reported for the analysed intervention arm.	
11	Comparator / control condition and co-interventions	Active comparator: app-based ePRO monitoring without real-time triage or care-team escalation; both arms received app-based monitoring and educational content. Rationale: clinical equipoise — the incremental value of triage over structured digital support was not established.	Methods — Intervention and comparator (control arm); Introduction; Discussion — Limitations
X (data security/privacy)	Data security and confidentiality	Patient data were handled in accordance with the GDPR and the German Federal Data Protection Act; all participants gave written informed consent; privacy and confidentiality were maintained; trial data were stored and managed on a secured cloud infrastructure	Methods — Ethical Considerations; Intervention and comparator

		operated by the developer partners.	
X26	Ethics approval	Central approval from the Ethics Committee of the Medical Faculty, Heidelberg University (S-658/2020), plus local approvals at the recruiting centres (Tuebingen 896/2020BO2; Mannheim 2021-544); conducted per the Declaration of Helsinki; written informed consent obtained. The ethics committee approvals themselves are provided with this revision; each lists the documents submitted to it, with version and date.	Methods — Ethical Considerations; Trial design
X27	Conflict of interest / relationship of developers to the intervention	Several authors (TMD, AB, MW) contributed to the development, content, and IT administration of the ENABLE application and the PIA platform (provided by the Center for Innovative Care and the Institute of Women's Health GmbH); this developer relationship to the evaluated intervention is disclosed.	Conflicts of Interest; Authors' Contributions

---

What changed against the R1 checklist

Subitem	R1	R2
Manuscript title	wrong — carried the pre-ED-1 title	corrected to the submitted title
Revision	R1	R2
1a-iii	"Web-/app-based … (smartphone/tablet/computer)" — ambiguous, which is what reviewer DX queried	native smartphone app (iOS/Android), PIA platform web-based (confirmed by the author team, 6 Aug 2026)
6	"Adoption was operationalized as sustained ePRO completion…"	completion of outcome assessments separated from intervention delivery; the term "adoption" removed (reviewer DX)
X26	ethics votes only	+ ethics approvals uploaded as supplementary files instead of a new 2026 supporting statement
11	rationale stated briefly	rationale sharpened (incremental value over structured digital support)
Location pointers	"Methods — Interventions", "Methods — Participants"	CONSORT topic names per the ED2-4 patch

⚠️ X26 depends on uploading the ethics approvals as supplementary files. The Heidelberg first approval S-658/2020 still has to be added to the final upload package; Mannheim approval dated 16 March 2021 is already available according to Marcus. Do not submit this checklist unless the referenced approvals are attached.

Heidelberg ethics committee on 6 August 2026 and had not arrived when this checklist was

prepared. If it is not enclosed at upload, this sentence must be removed or reworded — do not

submit a checklist claiming a document that is not in the package.

Checklist items based on: Eysenbach G; CONSORT-EHEALTH Group. CONSORT-EHEALTH: improving and standardizing evaluation reports of web-based and mobile health interventions. J Med Internet Res. 2011;13(4):e126. doi:10.2196/jmir.1923 (Open Access, CC BY).
