# Supplementary material for: Real-Time Electronic Patient-Reported Outcome Monitoring With Digital Triage and Care-Team Integration in Early Breast Cancer: ENABLE Randomized Controlled Trial
Source: J Med Internet Res. 2026 Oct 28;28:e95155. doi: 10.2196/95155 (PMC13717432; doi:10.2196/95155)
Supplement: Supplementary file 3 [file jmir-v28-e95155-s001.docx]

**Table S1. Response formats and scales used for assessing PRO-CTCAE**

| **Symptom (AE)** | **Item Wording** | **Response Format** | **CTCAE Anchor** | **AE Category (NIH National Cancer Institute)** |
| --- | --- | --- | --- | --- |
| Arm swelling/edema | Do you have swelling in your arms? | No (0), Mild (1), Severe (2) | Severity | Cardio/Circulatory |
| Leg swelling/edema | Do you have swelling in your legs? | No (0), Mild (1), Severe (2) | Severity | Cardio/Circulatory |
| Rash | Do you have a skin rash? | No (0), Mild (1), Severe (2), All over body (3) | Severity/Extent | Cutaneous |
| Hair loss | Do you have hair loss? | No (0), Hair falls out singly (1), In tufts (2) | Quantity/Pattern | Cutaneous |
| Nail changes | Do you have nail changes? | No (0), Mild (1), Severe (2) | Severity | Cutaneous |
| Wound healing disorder | Do you have a wound healing disorder? | No (0), Mild (1), Severe (2) | Severity | Cutaneous |
| Abdominal pain | Do you have abdominal pain? | No (0), Mild (1), Severe (2), Worst possible (3) | Severity | Gastrointestinal |
| Stomach pain | Do you have stomach pain? | No (0), Mild (1), Severe (2), Worst possible (3) | Severity | Gastrointestinal |
| Nausea | Do you suffer from nausea? | No (0), Mild (1), Severe (2), Worst possible (3) | Severity | Gastrointestinal |
| Vomiting | Do you suffer from vomiting? | No (0), 1–2 times/day (1), 3–5 times/day (2), >5 times/day (3) | Frequency | Gastrointestinal |
| Diarrhea | Do you have diarrhea? | No (0), Up to 3 stools/day more than normal (1), 4–6 more than normal (2), ≥7 more than normal (3) | Frequency | Gastrointestinal |
| Constipation | Do you suffer from constipation? | No (0), Mild (1), Severe (2) | Severity | Gastrointestinal |
| Appetite loss | Do you suffer from appetite loss? | No (0), Mild (1), Severe (2), Worst possible (3) | Severity | Gastrointestinal |
| Dysuria (burning on urination) | Do you have burning on urination? | No (0), Mild, intermittent (1), New onset (2), Cannot urinate despite urge, in pain (3) | Severity/Acute | Genitourinary |
| Fever | Do you have fever? | No (0), Elevated temp. ≤38°C (1), 38.1–38.5°C (2), >38.5°C or >38°C for >24h (3) | Severity/Temp | Miscellaneous |
| Chills | Do you have chills? | No (0), Yes, but no longer (1), Yes, currently (2) | Presence/Severity | Miscellaneous |
| Night sweats | Do you suffer from night sweats? | No (0), Occasionally (1), Drenched at night (2) | Frequency/Severity | Miscellaneous |
| Hot flashes | Do you have hot flashes? | No (0), Mild (1), Severe (2) | Severity | Miscellaneous |
| Breast pain | Do you have pain in your breast? | No (0), Mild (1), Severe (2), Worst possible (3) | Severity | Miscellaneous |
| Dry mucosa | Do you have dry mucosa? | No (0), Mild (1), Severe (2) | Severity | Miscellaneous |
| Bleeding/bruising | Do you have bruising or bleeding? | No (0), Mild bruising (1), Spontaneous nosebleed (2), Severe bleeding (3) | Severity/Type | Miscellaneous |
| Depression | Do you feel depressed? | No (0), Rarely (1), Often (2), Always (3) | Frequency | Mood |
| Finger tingling | Do you have tingling in your fingers? | No (0), Mild (1), Severe (2) | Severity | Neurological |
| Toe tingling | Do you have tingling in your toes? | No (0), Mild (1), Severe (2) | Severity | Neurological |
| Polyneuropathy of hands | Do you have sensory loss in your hands? | No (0), Mild (1), Occasionally drop things (2), Unable to button (3) | Function/Severity | Neurological |
| Polyneuropathy of foot | Do you have sensory loss in your feet? | No (0), Mild (1), Occasionally stumble (2), Unable to walk (3) | Function/Severity | Neurological |
| Cramps | Do you have cramps in arms or legs? | No (0), Mild (1), Severe (2) | Severity | Neurological |
| Dizziness | Do you suffer from dizziness? | No (0), Mild (1), Severe (2), Severe, new onset, cannot stand (3) | Severity/Acute | Neurological |
| Sore throat | Do you have a sore throat? | No (0), Mild (1), Severe (2), Worst possible (3) | Severity | Oral |
| Oral mucositis | Do you have oral mucositis? | No (0), Mild (1), Pain when eating (2), Cannot eat (3) | Severity/Function | Oral |
| Dry mouth | Do you have dry mouth? | No (0), Mild (1), Discomfort when eating (2), Cannot eat (3) | Severity/Function | Oral |
| Dysphagia (swallowing disorder) | Do you have difficulty swallowing? | No (0), Mild (1), Discomfort when eating (2), Cannot eat (3) | Severity/Function | Oral |
| Finger pain | Do you have pain in your fingers? | No (0), Mild (1), Severe (2), Worst possible (3) | Severity | Pain |
| Toe pain | Do you have pain in your toes? | No (0), Mild (1), Severe (2), Worst possible (3) | Severity | Pain |
| Palm pain | Do you have pain in your palm? | No (0), Mild (1), Severe (2), Worst possible (3) | Severity | Pain |
| Sole pain | Do you have pain in the sole of your foot? | No (0), Mild (1), Severe (2), Worst possible (3) | Severity | Pain |
| Headache | Do you have headaches? | No (0), Mild (1), Severe (2), Worst possible (3) | Severity | Pain |
| Back pain | Do you have back pain? | No (0), Mild (1), Severe (2), Worst possible (3) | Severity | Pain |
| Joint pain | Do you have joint pain? | No (0), Mild (1), Severe (2), Worst possible (3) | Severity | Pain |
| Limb pain | Do you have limb pain? | No (0), Mild (1), Severe (2), Worst possible (3) | Severity | Pain |
| Muscle pain | Do you have muscle pain? | No (0), Mild (1), Severe (2), Worst possible (3) | Severity | Pain |
| Chest pain | Do you have chest pain? | No (0), Mild (1), Severe (2), Worst possible (3) | Severity | Pain |
| Shortness of breath | Do you suffer from shortness of breath? | No (0), After one flight stairs (1), Less than one flight (2), At rest (3) | Severity/Function | Respiratory |
| Cough | Do you have a cough? | No (0), Mild (1), Severe (2), Coughing blood (3) | Severity/Alarm | Respiratory |
| Fatigue | Do you suffer from fatigue/exhaustion? | No (0), Need more rest (1), Daily activities limited (2), Depend on help (3) | Function/Severity | Sleep/Wake |
| Visual disturbance | Do you have visual disturbances? | No (0), Mild (1), Severe (2), Only blurred vision (3) | Severity | Visual/Perceptual |
| Dry or watery eyes | Do you have dry or watery eyes? | No (0), Mild (1), Severe (2) | Severity | Visual/Perceptual |
| Taste disorder | Do you have taste disturbances? | No (0), Mild (1), Severe, new onset (2) | Severity/Onset | Visual/Perceptual |
| Anosmia (loss of smell) | Do you have loss of sense of smell? | No (0), Mild (1), Severe, new onset (2) | Severity/Onset | Visual/Perceptual |
| Tinnitus | Do you suffer from tinnitus (ringing in ears)? | No (0), Mild (1), Severe (2) | Severity | Visual/Perceptual |
| Hearing loss | Do you have hearing loss? | No (0), Mild (1), Severe, new onset (2) | Severity/Onset | Visual/Perceptual |
| Other symptoms | Do you have other complaints? | No (0), Mild (free text) (1), Severe (free text) (2), Worst (free text) (3) | Severity (Free text) | — |

****

**Table S2. R Packages Used and Citations**

| **Package** | **Reference** |
| --- | --- |
| broom | Robinson D, Hayes A, Couch S (2025). *broom: Convert Statistical Objects into Tidy Tibbles*. doi:10.32614/CRAN.package.broom, R package version 1.0.10. |
| dplyr | Wickham H, François R, Henry L, Müller K, Vaughan D (2023). *dplyr: A Grammar of Data Manipulation*. doi:10.32614/CRAN.package.dplyr, R package version 1.1.4. |
| emmeans | Lenth R, Piaskowski J (2025). *emmeans: Estimated Marginal Means, aka Least-Squares Means*. doi:10.32614/CRAN.package.emmeans, R package version 2.0.0. |
| forcats | Wickham H (2025). *forcats: Tools for Working with Categorical Variables (Factors)*. doi:10.32614/CRAN.package.forcats, R package version 1.0.1. |
| forestploter | Dayimu A (2025). *forestploter: Create a Flexible Forest Plot*. doi:10.32614/CRAN.package.forestploter, R package version 1.1.3. |
| ggplot2 | Wickham H (2016). *ggplot2: Elegant Graphics for Data Analysis*. Springer-Verlag New York. ISBN 978-3-319-24277-4. |
| ggstatsplot | Patil I (2021). “Visualizations with statistical details: The ‘ggstatsplot’ approach.” *Journal of Open Source Software*, *6*(61), 3167. doi:10.21105/joss.03167. |
| ggsurvfit | Sjoberg D, Baillie M, Fruechtenicht C, Haesendonckx S, Treis T (2025). *ggsurvfit: Flexible Time-to-Event Figures*. doi:10.32614/CRAN.package.ggsurvfit, R package version 1.2.0. |
| glue | Hester J, Bryan J (2024). *glue: Interpreted String Literals*. doi:10.32614/CRAN.package.glue, R package version 1.8.0. |
| gt | Iannone R, Cheng J, Schloerke B, Hughes E, Lauer A, Seo J, Brevoort K, Roy O (2025). *gt: Easily Create Presentation-Ready Display Tables*. doi:10.32614/CRAN.package.gt, R package version 1.1.0. |
| gtsummary | Sjoberg D, Whiting K, Curry M, Lavery J, Larmarange J (2021). “Reproducible Summary Tables with the gtsummary Package.” *The R Journal*, *13*, 570–580. doi:10.32614/RJ-2021-053. |
| here | Müller K (2025). *here: A Simpler Way to Find Your Files*. doi:10.32614/CRAN.package.here, R package version 1.0.2. |
| lme4 | Bates D, Mächler M, Bolker B, Walker S (2015). “Fitting Linear Mixed-Effects Models Using lme4.” *Journal of Statistical Software*, *67*(1), 1–48. doi:10.18637/jss.v067.i01. |
| lubridate | Grolemund G, Wickham H (2011). “Dates and Times Made Easy with lubridate.” *Journal of Statistical Software*, *40*(3), 1–25. |
| lsr | Navarro D (2015). *Learning statistics with R: A tutorial for psychology students and other beginners (Version 0.6)*. University of New South Wales, Sydney, Australia. R package version 0.5.1. |
| marginaleffects | Arel-Bundock V, Greifer N, Heiss A (2024). “How to Interpret Statistical Models Using marginaleffects for R and Python.” *Journal of Statistical Software*, *111*(9), 1–32. doi:10.18637/jss.v111.i09. |
| Matrix | Bates D, Maechler M, Jagan M (2025). *Matrix: Sparse and Dense Matrix Classes and Methods*. doi:10.32614/CRAN.package.Matrix, R package version 1.7-4. |
| nlme | Pinheiro J, Bates D, R Core Team (2025). *nlme: Linear and Nonlinear Mixed Effects Models*. doi:10.32614/CRAN.package.nlme, R package version 3.1-168. Pinheiro JC, Bates DM (2000). *Mixed-Effects Models in S and S-PLUS*. Springer, New York. doi:10.1007/b98882. |
| patchwork | Pedersen T (2025). *patchwork: The Composer of Plots*. doi:10.32614/CRAN.package.patchwork, R package version 1.3.2 |

****

**Table S3. Conditional Mixed Effects Model on PROMIS-29 (linear Regression coefficients and corresponding 95% CIs)**

| **Predictors** | **Physical Function** | **Anxiety** | **Depression** | **Fatigue** |
| --- | --- | --- | --- | --- |
| Intercept | 48.88 (47.94 - 49.81) | 57.57 (56.49 - 58.66) | 54.23 (53.28 - 55.17) | 48.98 (47.77 - 50.19) |
| RPA vs. Control: Baseline | -0.79 (-2.12 to 0.55) | 0.26 (-1.28 to 1.80) | 0.08 (-1.26 to 1.43) | 1.53 (-0.19 to 3.25) |
| Control: Visit 2 | -3.49 (-4.49 to -2.49) | -4.22 (-5.22 to -3.22) | -1.50 (-2.35 to -0.66) | 3.92 (2.71 to 5.13) |
| Control: Visit 3 | -5.44 (-6.46 to -4.42) | -4.04 (-5.06 to -3.02) | -0.74 (-1.60 to 0.11) | 6.13 (4.90 to 7.36) |
| Control: Visit 4 | -6.23 (-7.26 to -5.19) | -3.25 (-4.28 to -2.22) | -0.44 (-1.30 to 0.43) | 5.95 (4.71 to 7.20) |
| Control: Visit 5 | -5.42 (-6.48 to -4.37) | -2.78 (-3.84 to -1.73) | -0.72 (-1.61 to 0.17) | 5.23 (3.96 to 6.50) |
| Control: Visit 6 | -4.74 (-5.82 to -3.65) | -3.08 (-4.17 to -2.00) | -1.25 (-2.16 to -0.34) | 3.51 (2.20 to 4.82) |
| RPA vs. Control: Visit 2 | 0.30 (-1.13 to 1.73) | -1.26 (-2.69 to 0.16) | -0.40 (-1.60 to 0.79) | -2.28 (-4.00 to -0.56) |
| RPA vs. Control: Visit 3 | 1.46 (0.02 to 2.91) | -1.63 (-3.08 to -0.19) | -1.11 (-2.32 to 0.10) | -3.46 (-5.20 to -1.72) |
| RPA vs. Control: Visit 4 | 1.64 (0.17 to 3.11) | -1.84 (-3.30 to -0.38) | -0.89 (-2.11 to 0.34) | -2.61 (-4.38 to -0.84) |
| RPA vs. Control: Visit 5 | 0.89 (-0.61 to 2.38) | -2.58 (-4.07 to -1.08) | -1.14 (-2.40 to 0.11) | -2.09 (-3.89 to -0.29) |
| RPA vs. Control: Visit 6 | 1.33 (-0.22 to 2.88) | -2.10 (-3.65 to -0.55) | -1.95 (-3.25 to -0.65) | -3.90 (-5.77 to -2.03) |
| **Random effects** |  |  |  |  |
| σ^2^ | 26.81 | 26.44 | 18.61 | 38.69 |
| τ_00_ PatientID | 23.01 | 41.85 | 33.93 | 45.42 |
| N PatientID | 487 | 486 | 486 | 486 |
| Observations | 2325 | 2322 | 2322 | 2322 |
| Marginal R² | 6.5% | 4.9% | 1.3% | 3.9% |

****

**Table S3 (continued)**

| **Predictors** | **Sleep Disturbance** | **Social Participation** | **Pain Interference** | **General Pain Intensity in past 7 days** |
| --- | --- | --- | --- | --- |
| Intercept | 50.48 (49.36 to 51.59) | 50.06 (48.94 to 51.18) | 51.73 (50.54 to 52.93) | 2.18 (1.89 to 2.48) |
| RPA vs. Control: Baseline | 0.62 (-0.97 to 2.20) | -1.25 (-2.85 to 0.35) | 0.36 (-1.35 to 2.06) | 0.15 (-0.27 to 0.57) |
| Control: Visit 2 | -0.87 (-1.91 to 0.18) | -3.77 (-4.84 to -2.70) | -0.96 (-2.21 to 0.29) | 0.11 (-0.20 to 0.41) |
| Control: Visit 3 | 1.04 (-0.02 to 2.10) | -5.36 (-6.44 to -4.27) | 0.15 (-1.12 to 1.43) | 0.37 (0.06 to 0.67) |
| Control: Visit 4 | 1.88 (0.81 to 2.95) | -5.55 (-6.65 to -4.45) | 0.31 (-0.97 to 1.60) | 0.59 (0.28 to 0.90) |
| Control: Visit 5 | 1.17 (0.07 to 2.26) | -5.34 (-6.47 to -4.22) | 0.65 (-0.66 to 1.97) | 0.42 (0.10 to 0.74) |
| Control: Visit 6 | 1.44 (0.31 to 2.56) | -4.36 (-5.52 to -3.21) | 2.92 (1.56 to 4.27) | 0.71 (0.39 to 1.04) |
| RPA vs. Control: Visit 2 | -0.11 (-1.59 to 1.37) | 1.37 (-0.15 to 2.89) | 0.90 (-0.88 to 2.68) | 0.02 (-0.41 to 0.45) |
| RPA vs. Control: Visit 3 | -1.78 (-3.29 to -0.28) | 2.58 (1.04 to 4.12) | -1.21 (-3.01 to 0.60) | -0.32 (-0.76 to 0.11) |
| RPA vs. Control: Visit 4 | -1.94 (-3.46 to -0.42) | 2.25 (0.68 to 3.81) | -0.52 (-2.34 to 1.31) | -0.38 (-0.82 to 0.06) |
| RPA vs. Control: Visit 5 | -1.08 (-2.63 to 0.48) | 2.69 (1.10 to 4.28) | -0.39 (-2.26 to 1.47) | -0.09 (-0.54 to 0.36) |
| RPA vs. Control: Visit 6 | -1.82 (-3.43 to -0.20) | 2.84 (1.18 to 4.49) | -1.44 (-3.38 to 0.49) | -0.26 (-0.73 to 0.21) |
| **Random effects** |  |  |  |  |
| σ^2^ | 28.66 | 30.10 | 41.38 | 2.43 |
| τ_00_ PatientID | 43.83 | 43.04 | 39.91 | 2.48 |
| N PatientID | 486 | 486 | 486 | 486 |
| Observations | 2322 | 2321 | 2320 | 2320 |
| Marginal R² | 0.8% | 3.5% | 1.1% | 0.8% |

*Annotations: Linear mixed-effects (random intercept for PatientID) with fixed effects for group, visit (6 levels; baseline = Visit 1), and their interaction; Scale: PROMIS-29 T-scores (mean 50, SD 10 in the reference population) with exception of General Pain Intensity. Higher scores indicate better health for Physical Function and Social Participation, and worse symptoms for Anxiety, Depression, Fatigue, Sleep Disturbance, Pain Interference and General Pain Intensity; Estimates: Cells show β (95% CI) from the conditional model; Reference coding: Intercept = Control at baseline. “Control: Visit k” = change from Control baseline at visit k. “RPA vs Control: Baseline/Visit k” = between-arm difference (RPA − Control) at baseline/visit k. Random effects: τ₀₀ = between-patient variance; σ² = residual (within-patient) variance. n/observations: N PatientID = unique participants contributing ≥1 measure; Observations = person-visits used in the model. Adjustment/missingness: Analyses use available cases at each visit; confidence intervals are Wald type.*

**Table S4. Hazard Ratios for Time to Deterioration in PROMIS-29 Domains (Intervention vs. Control Group)**

|  | **HR** | **95% CI** | ***P* value** | **n** | **events** |
| --- | --- | --- | --- | --- | --- |
| Physical Function | 0.87 | 0.67-1.12 | .27 | 390 | 238 |
| Anxiety | 0.69 | 0.45-1.07 | .10 | 388 | 85 |
| **Depression** | 0.66 | **0.44-0.98** | **.04** | 390 | 102 |
| **Fatigue** | 0.69 | **0.54-0.89** | **.004** | 382 | 261 |
| Sleep Disturbance | 0.85 | 0.64-1.12 | .25 | 380 | 195 |
| **Social Participation** | 0.76 | **0.59-0.97** | **.03** | 383 | 256 |
| Pain Interference | 0.96 | 0.72-1.27 | .76 | 382 | 193 |

*Annotations: Model: Cox proportional hazards for time to first deterioration; intervention = RPA, reference = Control; Scale/interpretation: HR < 1 favors RPA (lower hazard of deterioration); Event/censoring: Deterioration defined per protocol (see Methods); participants without an event were censored at last available assessment; n / events: n = participants included in the domain-specific model; events = number with ≥1 deterioration; Inference: Two-sided p values; 95% CIs from the Cox model*

**

**Table S5. PSQ-18 in comparison between intervention (RPA) and control group (non-RPA).** *Pairwise group comparisons of PSQ-18 subscales (exploratory P values not adjusted for multiplicity)*

| **Subscale** | **Control** **(non-RPA; n=172)** | | **Intervention** **(RPA, n=157)** | | ***P* value** |
| --- | --- | --- | --- | --- | --- |
|  | **Mean** | **SD** | **Mean** | **SD** |  |
| Total Score | 71.5 | 11.14 | 72.5 | 10.37 | 0.533 |
| General Satisfaction Score | 4.1 | 0.82 | 4.1 | 0.77 | 0.975 |
| Technical Quality Score | 4.2 | 0.68 | 4.2 | 0.66 | 0.456 |
| Interpersonal Manner Score | 4.4 | 0.69 | 4.4 | 0.61 | 0.492 |
| Communication Score | 4.0 | 0.82 | 4.1 | 0.74 | 0.701 |
| Management of Adverse Events Score | 4.1 | 0.80 | 4.1 | 0.77 | 0.483 |
| Time Spent with the Doctor | 3.8 | 0.90 | 3.8 | 0.90 | 0.708 |
| Accessibility and Convenience Score | 3.6 | 0.76 | 3.7 | 0.69 | 0.167 |

*Annotations: Instrument/scale: PSQ-18 subscales scored 1–5; higher scores indicate greater satisfaction. Total score rescaled to 0–100; Display/statistics: Mean (SD) by arm; two-sided between-group P values from Wilcoxon rank-sum tests; exploratory, no multiplicity adjustment; n: Participants with available PSQ-18 at the prespecified assessment time point; Missingness: Analyses use available cases.*

**

**Table S6. PSQ-18 in comparison between neoadjuvant and adjuvant therapy.** *Pairwise group comparisons of PSQ-18 subscales (exploratory P values not adjusted for multiplicity)*

| **Subscale** | **Neoadjuvant (n=236)** | | **Adjuvant (n=66)** | | ***P* value** |
| --- | --- | --- | --- | --- | --- |
|  | **Mean** | **SD** | **Mean** | **SD** |  |
| Accessibility and Convenience Score | 3.7 | 0.71 | 3.5 | 0.64 | 0.044 |
| Time Spent with the Doctor | 3.8 | 0.90 | 3.6 | 0.81 | 0.018 |
| Management of Adverse Events Score | 4.1 | 0.75 | 4.0 | 0.76 | 0.126 |
| Communication Score | 4.1 | 0.77 | 3.9 | 0.77 | 0.046 |
| Interpersonal Manner Score | 4.4 | 0.64 | 4.3 | 0.60 | 0.075 |
| Technical Quality Score | 4.2 | 0.67 | 4.0 | 0.66 | 0.010 |
| General Satisfaction Score | 4.2 | 0.77 | 3.9 | 0.77 | 0.003 |
| Total Score | 72.9 | 10.51 | 69.4 | 9.98 | 0.013 |

*Annotations: See Table S5*

**

**Table S7. PSQ-18 in comparison between pCR and non-pCR among patients with neoadjuvant therapy.** *Pairwise group comparisons of PSQ-18 subscales (exploratory P values not adjusted for multiplicity)*

| **Subscale** | **Non-pCR (n=110)** | | **pCR** **(n=109)** | | ***P* value** |
| --- | --- | --- | --- | --- | --- |
|  | **Mean** | **SD** | **Mean** | **SD** |  |
| Accessibility and Convenience Score | 3.8 | 0.76 | 3.6 | 0.68 | 0.141 |
| Time Spent with the Doctor | 3.8 | 0.93 | 3.8 | 0.88 | 0.906 |
| Management of Adverse Events Score | 4.1 | 0.79 | 4.2 | 0.73 | 0.222 |
| Communication Score | 4.0 | 0.88 | 4.1 | 0.66 | 0.822 |
| Interpersonal Manner Score | 4.4 | 0.71 | 4.5 | 0.59 | 0.346 |
| Technical Quality Score | 4.2 | 0.68 | 4.3 | 0.67 | 0.188 |
| General Satisfaction Score | 4.1 | 0.73 | 4.2 | 0.78 | 0.479 |
| Total Score | 72.5 | 11.48 | 73.2 | 9.67 | 0.606 |

*Annotations: See Table S5*

****

**Figure S1. Kaplan–Meier curves for time to complete ePRO discontinuation by trial arm.**

*Annotations Shown is the probability of remaining under ePRO monitoring over time in the control and RPA groups. The event was defined as complete ePRO discontinuation, that is, the first missed scheduled assessment from which all subsequent questionnaires remained unanswered. Patients who continued to complete at least one questionnaire until the end of follow-up were right-censored at their last observation. Shaded areas indicate 95% confidence intervals. The arm effect was estimated using a univariable Cox proportional-hazards model.*****

**Figure S2. PSQ-18 total and subscale scores comparing neoadjuvant (n=236) versus adjuvant (n=66) therapy.**

*Annotations: Box plots indicate median and quartiles, yellow diamonds mark group means, violin plots show density distributions in both groups.*

**Figure S3. PSQ-18 total and subscale scores comparing non-pCR (n=110) versus pCR (n=109) patients under neoadjuvant therapy.**

*Annotations: Box plots indicate median and quartiles, yellow diamonds mark group means, violin plots show density distributions in both groups.*
